# Tiara Ni Clusters for Electrocatalytic Nitrate Reduction to Ammonia with 97% Faradaic Efficiency

**DOI:** 10.1021/jacs.5c04950

**Published:** 2025-06-20

**Authors:** Xinrui Gu, Jingjing Zhang, Song Guo, Yifei Zhang, Liangliang Xu, Rongchao Jin, Gao Li

**Affiliations:** † School of Chemistry and Chemical Engineering, 71203 Inner Mongolia Normal University, Hohhot 010018, China; ‡ Dalian Institute of Chemical Physics, Chinese Academy of Sciences, Dalian 116023, China; Δ Department of Chemistry, University of Puerto Rico, Rio Piedras, San Juan, Puerto Rico 00931, United States; δ Department of Chemistry, 6612 Carnegie Mellon University, Pittsburgh, Pennsylvania 15213, United States; $ University of Chinese Academy of Sciences, Beijing 100049, China

## Abstract

The electroreduction of nitrate (NO_3_^–^) for sustainable ammonia (NH_3_) production has recently emerged as a green process to solve water contamination and produce valuable chemicals. In this study, we developed Ni_6_@CuFe-LDH composites comprising tiara Ni_6_(SC_2_H_4_COOH)_12_ (Ni_6_) clusters anchored on the edges of 2D CuFe-LDH (LDH: layered double hydroxides) nanosheets via electrostatic interactions. The Ni_6_@CuFe-LDH catalyst exhibits high electrochemical performance in nitrate reduction reaction (NO_3_RR). Specifically, the Ni_6_@CuFe-LDH gives rise to an excellent faradaic efficiency of ∼97%, significantly surpassing the ∼73% FE of the pristine CuFe-LDH, with the NH_3_ productivity (0.91 mmol mg^–1^ h^–1^) being similar to that of the CuFe-LDH. Mechanistic studies reveal that the superior electrocatalysis of Ni_6_-based catalysts is primarily due to the synergistic interaction between Ni_6_ clusters and CuFe-LDH, which alters the rate-determining step (RDS) of the desorption of *NH_3_ species (for CuFe-LDH) to the *NO_3_ → *NO_2_ step (for Ni_6_@CuFe-LDH); this is corroborated by the control experiments of NO_2_RR, in situ Raman and infrared spectroscopies, and computational approaches. In all, these efforts push forward the NO_3_RR research to study the structure–property relationships from the micro/nano-level to the precise atomic-level.

## Graphical abstract

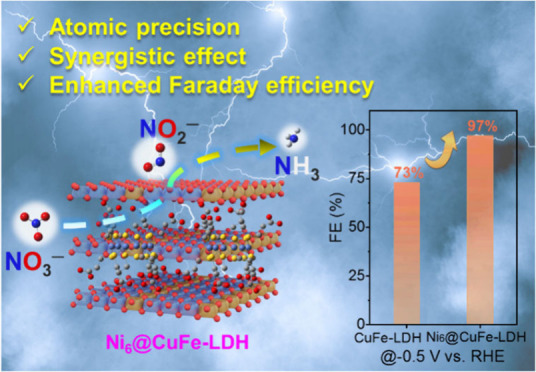

## 1 Introduction

Ammonia (NH_3_) is one of the most widely produced inorganic compounds and plays a crucial role in the chemical industry, fertilizer and pharmaceutical production, as well as other fields.^1,2^ The Haber-Bosch ammonia synthesis process developed in the early 20th century is still the main route for NH_3_ production. However, this process requires harsh reaction conditions of high temperature (350–450 °C) and high pressure (150–200 bar), leading to excessive energy consumption and CO_2_ emission.^3,4^ These drawbacks are particularly problematic in light of the current energy-saving trend and the growing concerns over climate changes.^5–7^ Therefore, it is crucial to develop a clean, efficient and sustainable NH_3_ production method. The electrochemical nitrate reduction reaction (NO_3_RR) under ambient conditions offers a promising alternative, serving as an effective energy storage solution that can harness intermittent renewable energy sources, such as solar and wind power.^8–10^ Furthermore, NO_3_RR can reduce nitrate (as a pollutant) to NH_3_, providing dual benefits of waste valorization and resource recovery.^11^

The electrochemical reduction of NO_3_^–^ to NH_3_ is a complex process that involves the transfer of eight electrons and multiple steps of hydrogenation and deoxidation (NO_3_^–^ + 6H_2_O + 8e^–^ → NH_3_ + 9OH^–^).^12–14^ This multistep process involves various intermediates and reaction pathways. To develop efficient NO_3_RR catalysts, three key parameters must be optimized: (i) the selectivity of NO_3_RR over the hydrogen evolution reaction (HER), (ii) high faradaic efficiency in the overall process, and (iii) high ammonia productivity with good stability of catalysts.

Currently, Cu-based catalysts are considered to be one of the most promising NO_3_RR catalysts due to the similarity between the highest occupied d-orbital of Cu and the lowest unoccupied π*-orbital of NO_3_^–^ anions.^15^ Recently, it has been reported that a Fe/Cu composite catalyst supported on holey nitrogen-doped graphene exhibits a NH_3_ formation rate of 1.08 mmol h^–1^ mg^–1^ with an ammonia FE of ∼60% at −0.5 V vs RHE.^16^ Although copper composites show excellent performance in NO_3_^–^ adsorption and conversion to NO_2_^–^ species, the further reduction to NH_3_ product remains inefficient and is prone to inactivation during the long-term operation.^17,18^ To address these challenges, various strategies have been developed, such as crystal face engineering, alloying, oxidation, doping modification, and single-atom dispersion strategies, to improve the intrinsic FE for NH_3_ production over Cu-based catalysts.^19^ In addition, layered double hydroxides (LDHs) have gained increasing attention in electrochemistry due to the advantages including low cost, facile synthesis, tunable electronic structure and morphological characteristics, as well as excellent electron transfer capabilities.^20,21^

On the other hand, atomically precise nickel nanoclusters (Ni_*n*_(SR)_2*n*_) have garnered interest in electrocatalysis due to their unique electronic properties, with the reported reactions including oxygen evolution reaction (OER), electrocatalytic nitrogen reduction to ammonia, etc.^22–24^ Recently, Maman and co-workers reported that the phenylethanethiolated nickel clusters can electrocatalytically reduce N_2_ gas to ammonia with a 25% FE.^25^ These findings inspired us to explore the electrochemical nitrate reduction using Ni_6_(SC_2_H_4_COOH)_12_ clusters. Its well-defined structure can also be explored as a well-defined model system to study structure–activity relationships. Herein, we prepared Ni_6_@CuFe-LDH composites via electrostatic interactions for electrocatalytic nitrate reduction to ammonia, Scheme 1. A high FE of ∼97% with a NH_3_ productivity of 0.91 mmol mg^–1^ h^–1^ at −0.5 V vs RHE was achieved by the cooperativity between Ni_6_(SC_2_H_4_COOH)_12_ and 2D CuFe-LDH nanosheets. The experimental results, together with theoretical calculations, have elucidated the mechanism (especially the much improved FE) of NO_3_RR by nickel clusters at the atomic/molecular-level.

**Scheme 1:**
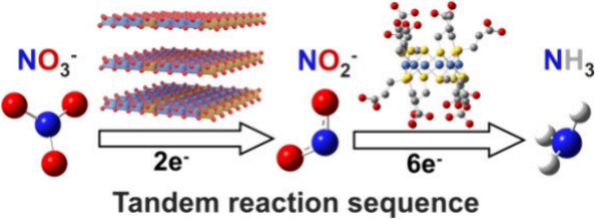
Tandem Reactions of Electrocatalytic Nitrate Reduction to Ammonia over Ni_6_@CuFe-LDH Electro-catalysts

## 2 Materials and Methods

### 2.1 Preparation of Ni_6_@CuFe-LDH

The Ni_6_@CuFe-LDH composite was prepared using a modified coprecipitation protocol.^26^ Three aqueous solutions were first prepared: solution A containing hexamethylenetetramine (HMTA), solution B containing Fe­(NO_3_)_3_ and Cu­(NO_3_)_2_, and solution C containing Ni_6_(SC_2_H_4_COOH)_12_ (hereafter abbreviated as Ni_6_). Solutions of B and C were simultaneously added into solution A under vigorous stirring at 60 °C. The resulting suspension was then aged at 60 °C for 2 h without stirring. Afterward, the solids of Ni_6_@CuFe-LDH composites were collected by filtration and washed with deionized water until the supernatant reached a pH of ∼7. For comparison, CuFe-LDH was prepared using the same procedure by using solutions A and B only.

Additionally, Ni_6_/CuFe-LDH and Ni_6_/AC (AC: activated carbon) were prepared by the simple physical adsorption method. CuFe-LDH or AC was ultrasonically treated in water, the Ni_6_ cluster solution was then added dropwise to the pretreated substrate suspension under continuous stirring, and the mixture was heated at 60 °C for 2 h. Subsequently, the product was washed with water/ethanol to remove unbound clusters, and dried at 80 °C.

### 2.2 NO_3_RR Electrochemistry

The electrochemical NO_3_RR measurements were recorded on a CHI 760E workstation at ambient temperature. To prevent the oxidation of cathodic product (NH_3_) at the anode, a typical three-electrode H-cell separated by a proton-exchange membrane (Nafion 117) was employed. The cell configuration included the as-prepared samples (working electrode, WE), Hg/HgO (reference electrode, RE), and graphite rod (counter electrode, CE). Each cell contained 40 mL of electrolytes (1 M KOH or 1 M KOH + 0.1 M KNO_3_). Before tests, the catalyst ink was prepared by dispersing samples into a mixture of Nafion, water and ethanol via ultrasonication for 30 min. The ink was then coated on carbon paper (CP, 0.5 × 0.5 cm^2^) to make a 0.6 mg_cat_ cm^–2^ loading.

The electrochemical tests were repeated more than three times, with error bars representing the standard deviation of the data. All potentials were referenced to the reversible hydrogen electrode (RHE) according to the equation: E_RHE_= E_Hg/HgO_ + 0.098 V + 0.059 × pH. Linear sweep voltammetry (LSV) was conducted from 0.1 V to –0.5 V vs RHE with a scan rate of 5 mV s^–1^. In addition, *iR* compensation (90%) and stirring (1600 rpm) were performed during the LSV test. Then, the potentiostatic tests were carried out at the potential range from 0 V to –0.5 V for 1 h to evaluate the NH_3_ formation rate and FE. The double-layer capacitance (C_dl_) was determined via cyclic voltammetry (CV) at different scan rates between 0.4 and 0.5 V vs RHE. Electrochemical impedance spectroscopy (EIS) was measured at 0 V vs RHE over a frequency range from 10^–2^ to 10^5^ Hz. The electrochemical active surface area (ECSA) was calculated by the equation: $\mathrm{ECSA}=\frac{C_{\mathrm{dl}}}{60\;\mu F\;{\mathrm{cm}}^{-2}}{\mathrm{cm}}_{\mathrm{ECSA}}^{2}$ .

## 3 Results and Discussion

### 3.1 Characterization of Ni_6_@CuFe-LDH Composites

The Ni_6_@CuFe-LDH composite was synthesized via a coprecipitation method, which was based on the electrostatic interactions between the deprotonated carboxylic acid group of MPA (3-mercaptopropionic acid) ligands and Cu/Fe cations. The preparation process was shown in Figure 1a. First, Ni_6_ clusters were synthesized according to a previous method^23^ and confirmed by the cluster’s characteristic spectral features using UV–vis spectroscopy (Figure S1). Specifically, the free Ni_6_ clusters in an aqueous solution show three characteristic absorption bands at 336, 406, and 547 nm in the UV–vis spectrum. These peaks correspond to ligand-to-metal charge-transfer, metal-centered interband transition and ligand-centered transition, respectively.^27^ Subsequently, the Ni_6_@CuFe-LDH composite was prepared by electrostatic interactions of Ni_6_ clusters with LDH in a coprecipitation process. The XRD analysis (Figures 1b and S2) elucidates the structural evolution across the catalyst series, where CuFe-LDH demonstrates a highly ordered crystalline lattice evidenced by characteristic (003), (006), (012), (015), and (018) diffraction planes at 12.8°, 25.8°, 33.6°, 36.5°, and 43.6°, respectively, which are perfectly aligned with the standard pattern of layered double hydroxide framework (PDF #50-0235). The persistence of these characteristic reflections in both Ni Ni_6_/CuFe-LDH (prepared by simple physical mixing) and Ni_6_@CuFe-LDH (prepared by coprecipitation) underscores the retention of the parent LDH’s structural fidelity following the cluster integration, while the complete absence of extraneous NiO or NiS phases in Ni_6_@CuFe-LDH conclusively verifies the preservation of Ni_6_ cluster integrity under mild synthetic conditions. This phase purity stands in stark contrast to Ni_6_/AC, which exclusively manifests broad amorphous carbon signatures (24° and 43°), confirming the noncrystalline nature of AC-supported clusters.^28^ TEM analysis coupled with elemental mapping systematically compares the morphologies of Ni_6_/AC, CuFe-LDH, Ni_6_/CuFe-LDH, and Ni_6_@CuFe-LDH. Ni_6_/AC exhibits uniformly dispersed clusters (∼1 nm diameter) anchored on the AC substrate, while CuFe-LDH adopts its characteristic nanosheet-like structure (Figures S3 and S4). In contrast, Ni_6_/CuFe-LDH (i.e., prepared via physical mixing) shows severe cluster aggregation on the LDH surface, highlighting interfacial incompatibility (Figure S5). Remarkably, Ni_6_@CuFe-LDH demonstrates homogeneous cluster distribution across the LDH matrix, confirming the success of the electrostatic anchoring strategy in achieving structural uniformity. Furthermore, linear scanning profiles across the catalyst cross-section reveal homogeneous elemental distribution (Ni, Cu, Fe) at the atomic scale, confirming effective interfacial integration (Figures 1c,d and S6).

**Figure 1:**
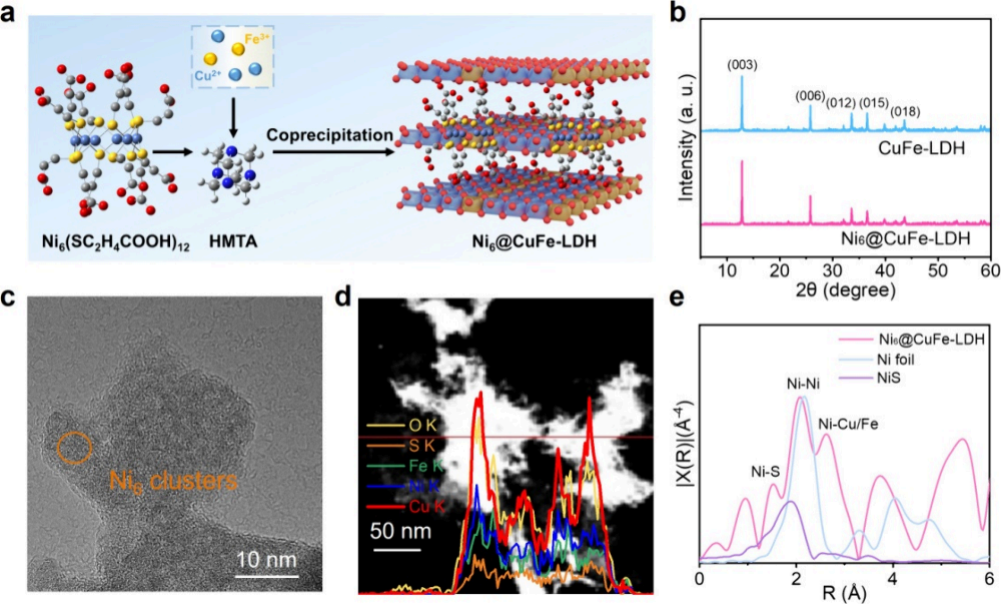
(a) Schematic illustration of the preparation procedure of Ni_6_@CuFe-LDH composites. (b) XRD patterns of CuFe-LDH and Ni_6_@CuFe-LDH. (c) TEM image of the Ni_6_@CuFe-LDH. (d) Linear-scanning profiles of the cross-section of Ni_6_@CuFe-LDH. (e) Ni K-edge EXAFS spectra for Ni_6_@CuFe-LDH and the standard references.

The XPS analysis systematically elucidates the electronic states and interfacial interactions in Ni_6_/AC, CuFe-LDH, Ni_6_/CuFe-LDH, and Ni_6_@CuFe-LDH. For Ni_6_/AC, the Ni 2p_3/2_ peaks at 855.3 eV (Ni 2p_3/2_) and 873.3 eV (Ni 2p_1/2_) with satellite features at 860.9/879.4 eV confirm Ni–S coordination, while S 2p_3/2_ at 163.8 and 168.7 eV corresponds to sulfur in oxidized states (e.g., sulfates) (Figure S7).^29,30^ In CuFe-LDH, Cu 2p_3/2_ at 934.8 eV (Cu^2+^) with satellites at 941.7–962.7 eV and Fe 2p_3/2_ at 711.1 eV (Fe^3+^) with satellites at 719.5–731.6 eV validate the pristine LDH structure (Figure S8).^31,32^ Ni_6_/CuFe-LDH retains Ni 2p_3/2_ at 855.5 eV and S 2p_3/2_ at 163.6 eV, identical to Ni_6_/AC, while Cu/Fe peaks (934.8 eV Cu^2+^; 711.3 eV Fe^3+^) mirror CuFe-LDH, confirming negligible interfacial interactions (Figure S9).^33^ Strikingly, Ni_6_@CuFe-LDH exhibits a Ni 2p_3/2_ upshift to 856.5 eV, S 2p_3/2_ broadening (163.8/165.0 eV), Cu 2p_3/2_ downshift to 934.5 eV, and Fe 2p_3/2_ stabilization at 711.3 eV, collectively indicating covalent Ni–O–Cu/Fe bonding via electrostatic anchoring (Figure S10). The O 1s spectrum further reveals lattice oxygen (O_l_, 530.2 eV) dominance (36.2% vs 16.4% in CuFe-LDH), confirming −COO-Cu/Fe interfacial bonds (Figure S11 and Table S1).^34,35^

To elucidate the detailed electronic structure and coordination environment of Ni_6_@CuFe-LDH, we performed X-ray absorption spectroscopy (XAS) studies.^36^ For comparison, a set of reference samples of Ni foil and NiS were measured. In the X-ray absorption near-edge structure (XANES) spectrum of Ni K edge, the white line intensity of Ni_6_@CuFe-LDH was higher than that of NiS, indicating a higher oxidation state of Ni species and suggesting partial electron transfer from Ni_6_ to neighboring CuFe-LDH (Figure S12), which is consistent with the preceding Ni 2p XPS results.^37^ The EXAFS analysis elucidates the hierarchical coordination environment and interfacial interactions governing the catalytic system (Figure 1e). The first peak at 1.52 Å corresponds to the first coordination shell of Ni, involving Ni–S bonds or Ni–O bonds from surface hydroxyls of the CuFe-LDH support.^38^ The sulfur ligands, acting as strong σ-donors, may induce an upward shift in the Ni d-band center, enhancing intermediate adsorption. The second peak at 2.08 Å arises from Ni–Ni metallic bonding, indicating the presence of a metallic core within the Ni_6_ clusters. The incorporation of Fe via Fe–O–Ni bridging likely optimizes charge redistribution, lowering the energy barrier for intermediate formation. The third peak at 2.62 Å reflects long-range interactions between Ni and the CuFe-LDH support, possibly involving Ni–Cu–O or Ni–Fe–O super exchange pathways or interfacial Jahn–Teller distortions (Table S2). This interfacial synergistic effect optimizes the adsorption/desorption equilibrium of key intermediates (such as *NO, *NH) in the NO_3_^–^ reduction reaction (NO_3_RR) through the synergistic electronic regulation of sulfur ligands and metal nuclei, thereby significantly enhancing catalytic selectivity. Overall, the XAS results reveal the multiscale coordination structure of Ni_6_@CuFe-LDH short-range Ni–S/O bonds regulate the electronic state, medium-range Ni–Ni/Fe interactions stabilize the cluster geometry, and long-range interinterface coupling ensures efficient charge transfer, providing direct experimental evidence for atomic-level active site design and reaction pathway optimization. To verify the electrostatic interaction between the clusters and the carrier LDH during the synthesis process, we conducted zeta potential measurements under the synthetic pH conditions (pH = 10). As shown in Table S3, Ni_6_(MPA)_12_ registered −12.38 mV, attributed to the negative surface charges of deprotonated MPA ligands, whereas CuFe-LDH exhibited +31.25 mV, aligning with its inherent cationic hydroxide layers. The composite Ni_6_@CuFe-LDH demonstrated a moderate potential of +17.76 mV, signifying partial charge neutralization between the components. The substantial difference, Δζ (43.63 mV), underscores the essential charge complementarity for Coulombic anchoring of Ni_6_ clusters onto LDH. The intermediate potential further reveals an equilibrium between the charge shielding efficacy and interfacial bond formation.

### 3.2 Electrochemical NO_3_RR Performance

The NO_3_RR performance of Ni_6_@CuFe-LDH was evaluated in a CHI 760E electrochemical workstation with a standard three-electrode system and H-type electrolyzer (Figure 2a). For comparison, Ni_6_/AC, CuFe-LDH and Ni_6_/CuFe-LDH were also investigated under the identical conditions. The quantification of NH_3_ and NO_2_^–^ products was performed by a colorimetric method (see the standard curves in Figure S13).^39^ In order to distinguish the contribution of NO_3_RR vs HER, we first performed linear sweep voltammetry (LSV) both with and without NO_3_^–^ in the electrolyte. The LSV curve shows that, at the same potential, the current density of the four catalysts in the electrolyte containing 1 M KOH and 0.1 M NO_3_^–^ is significantly higher than that in the electrolyte without NO_3_^–^, indicating a certain NO_3_^–^ activity (Figures 2b and S14).^40^ Notably, the current density of Ni_6_/AC is much lower than that of the other three catalysts, suggesting that Ni_6_ cluster itself exhibits weak catalytic activity toward NO_3_RR, and the contribution of Ni_6_ clusters in the Ni_6_@CuFe-LDH catalyst to NH_3_ generation in NO_3_RR is minimal.^41^ To further assess the NO_3_RR catalytic activity, chronoamperometry test was carried out by continuous electrolysis for 1 h over a potential range from 0 to −0.5 V. The NH_3_ formation rate and faradaic efficiency (FE) of NO_3_RR are shown in Figure 2c and 2d, respectively. The NH_3_ formation rate on Ni_6_@CuFe-LDH is similar to that of CuFe-LDH. At the potential of −0.5 V vs RHE, Ni_6_@CuFe-LDH achieves a yield of 0.91 mmol mg^–1^ h^–1^. Interestingly, the FE is drastically different between the two catalysts, with Ni_6_@CuFe-LDH exhibiting a FE of 96.8% and CuFe-LDH showing only 72.5%. Notably, CuFe-LDH exhibits a higher propensity for NO_2_^–^ accumulation during NO_3_RR, while comparative electrochemical analysis of Ni_6_@CuFe-LDH and CuFe-LDH reveals distinct HER suppression behavior. At −0.5 V vs RHE, CuFe-LDH demonstrates doubled HER current density, supported by Tafel analysis showing a lower Tafel slope (Figure S15). This electrochemical contrast underscores the Ni_6_ cluster’s dual functionality in simultaneously mitigating parasitic hydrogen evolution while optimizing nitrate conversion pathways. The yield over the Ni_6_/CuFe-LDH catalyst (prepared via a simple physical adsorption) was significantly lower than that of Ni^6^@CuFe-LDH and CuFe-LDH. The difference in activity between the two stems from the different interfacial bonding mechanisms. Ni_6_@CuFe-LDH is formed by in situ coprecipitation. The carboxylic acid groups (−COO^–^) of the ligands on Ni_6_ clusters bind to Cu/Fe–OH in LDH through covalent bonds, achieving strong electronic coupling. In contrast, Ni_6_/CuFe-LDH is prepared through physical adsorption. The clusters adhere to the substrate through weak van der Waals forces, resulting in aggregation (evidenced by TEM in Figure S5) and poor charge transfer. Besides, Ni_6_/AC exhibits a lower NH_3_ yield rate compared to other catalysts, this aligns with its reduced current density in LSV curves, reflecting slower reaction kinetics. However, Ni_6_/AC achieves an ideal FE, indicating that the Ni_6_ clusters intrinsically enhance selectivity toward NH_3_ synthesis. The data collectively emphasize that while Ni_6_ provides selective active sites, the electronic synergy with CuFe-LDH is critical for maximizing both activity and efficiency. Figure S16 summarizes the key performance parameters of the catalysts mentioned above. To eliminate potential interference from the electrocatalyst itself and external NH_3_ contamination, nitrate-free electrolysis experiments and open circuit potential (OCP) control experiments were conducted on Ni_6_@CuFe-LDH.^42^ The tests demonstrated that the ammonia generation was negligible (Figure 2e). Meanwhile, in order to trace the source of NH_3_ in the reaction, we conducted ^15^N isotope labeling experiments.^43^ ^1^H nuclear magnetic resonance (NMR) spectra were employed to distinguish ^14^NH_4_^+^ and ^15^NH_4_^+^ products.

**Figure 2:**
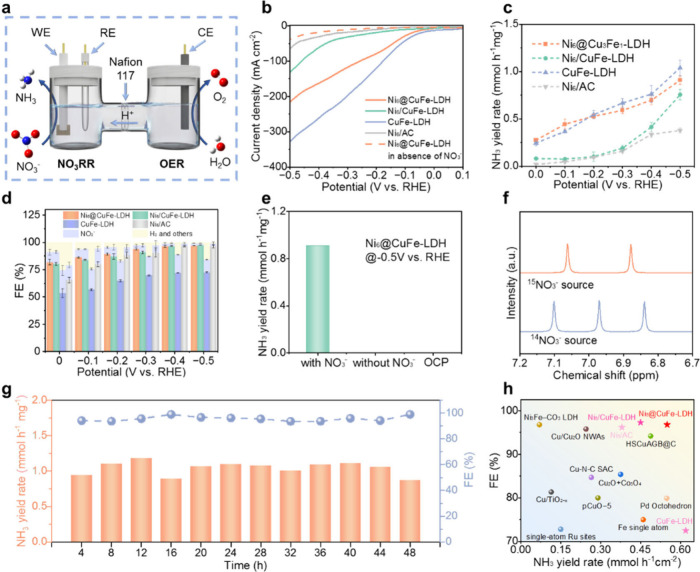
Electrocatalytic performance for NO_3_RR. (a) Schematic illustration of H-type electrolyzer. (b) LSV curves in 1 M KOH with and without 0.1 M NO_3_^–^ solutions. (c) NH_3_ yield rate and (d) FE of NO_3_RR for different samples at various potentials. (e) NO_3_RR performance of Ni_6_@CuFe-LDH with different conditions. (f) ^1^HNMR measurements of the electrolyte with ^15^NO_3_^–^ and ^14^NO_3_^–^ as the N sources over Ni_6_@CuFe-LDH. (g) Long-term chronoamperometry tests at −0.5 V vs RHE. (h) Comparison of the NO_3_RR performance with other catalysts reported in the literature. As illustrated in Figure 2f, the ^14^NH_4_^+^ produced using ^14^NO_3_^–^ as the feed N source exhibited characteristic triple peaks, while replacing ^14^NO_3_^–^ with ^15^NO_3_^–^ produced obvious double peaks, clearly confirming that NH_3_ was synthesized through the electrocatalytic reduction of NO_3_^–^.

Further, the double-layer capacitance (C_dl_) was measured to calculate the electrochemical active surface area (ECSA).^44^ It can be observed that the C_dl_ values of Ni_6_@CuFe-LDH and CuFe-LDH are obviously larger than that of Ni_6_/CuFe-LDH (Figures S17 and S18), which means that the Ni_6_ clusters on Ni_6_/CuFe-LDH, obtained by simple physical adsorption, are likely anchored on the active or defect sites of the LDH, with a strong interaction between Ni_6_ clusters and LDH. To gain further insight into the electrode kinetics during the NO_3_RR, we performed electrochemical impedance spectroscopy (EIS) at 0 V vs RHE with frequencies ranging from 10^–2^ Hz to 10^5^ Hz (Figure S19).^45^ Generally, a smaller semicircle diameter in the EIS spectrum indicates lower charge transfer resistance (R_ct_), and a smaller R_ct_ suggests better electron-transfer kinetics in electrocatalysis. It is worthy to note that the R_ct_ of Ni_6_@CuFe-LDH is similar to that of CuFe-LDH, but much smaller than that of Ni_6_/CuFe-LDH, which is consistent with the C_dl_ values. The turnover frequencies (TOF) of Ni_6_@CuFe-LDH, CuFe-LDH, and Ni_6_/AC catalysts were compared at –0.5 V vs RHE (Figure S20). The Ni_6_@CuFe-LDH catalyst exhibits a TOF of 0.225 s^–1^, significantly exceeding those of Ni_6_/AC (0.014 s^–1^) and CuFe-LDH (0.047 s^–1^). This marked enhancement in TOF underscores the synergistic interaction between the Ni_6_ clusters and the CuFe-LDH matrix. Such interfacial synergy likely facilitates enhanced active site exposure, optimizes the electronic structure to accelerate electron transfer, and stabilizes key reaction intermediates during the catalytic process. As a key factor in evaluating the catalyst performance, the long-term stability was assessed through cyclic and continuous tests at an optimal potential of –0.5 V. The chronopotentiometry measurements of Ni_6_@CuFe-LDH demonstrated stable current density and no obvious decline in NH_3_ rate and FE during 12 cycles (48 h), indicating cyclic stability and repeatability of the experiment, which exceeds most previously reported NO_3_RR catalysts, and long-term tests also show that the current density of the catalyst has not changed significantly (Figures 2g and S21–S23). Remarkably, the electrocatalytic performance of Ni_6_@CuFe-LDH for ammonia production compares favorably with other documented NO_3_RR electrocatalysts (Figure 2h and Table S4).

To investigate the structure of the material before and after the reaction, we characterized Ni_6_@CuFe-LDH by XRD, TEM, and XPS analyses. The structural evaluation of the postreaction Ni_6_@CuFe-LDH reveals selective modifications in the disappearance of the (003) basal plane reflection—a phenomenon attributed to partial interlayer restructuring through ligand-mediated stacking fault generation rather than bulk phase decomposition, as confirmed by the retained diffraction intensities of (006), (015), and (018) planes (Figure S24). High-resolution TEM characterization reveals that the material retains its intact lamellar structure characteristic of LDH, with Ni_6_ clusters showing no observable agglomeration (Figure S25). The used Ni_6_@CuFe-LDH shows a slight Ni 2p_3/2_ downshift (856.1 eV) and S 2p_3/2_ reduction to 162.3 eV, suggesting partial Ni^3+^→Ni^2+^ reduction and sulfur reconfiguration into Ni–S bonds. Cu 2p_3/2_ shifts to 934.0 eV (Cu^+^/Cu^2+^ mixture), while Fe 2p_3/2_ remains stable at 711.0 eV, underscoring the role of Fe^3+^ as an electron reservoir. In comparison, Ni_6_/CuFe-LDH lacks these interfacial trends, with Ni/S/Cu/Fe peaks nearly unchanged after the reaction (Figure S26).

To elucidate the distinct roles of Cu and Fe in CuFe-LDH, comparative NO_3_RR studies were conducted on Ni_6_@Cu-LDH and Ni_6_@Fe-LDH alongside the Ni_6_@CuFe-LDH (Figure S27), with the latter demonstrating superior NH_3_ yield and FE. The Cu-dominated system exhibited an enhanced catalytic activity due to the ability of Cu to facilitate nitrate adsorption via *NO_3_^–^–π antibonding interactions, while Fe stabilizes reaction intermediates through Fe–O coordination. Charge complementarity between the negatively charged Ni_6_ clusters and the cationic LDH framework enables synergistic interactions, as evidenced by zeta potential analysis. In addition, to explore the role of Cu and Fe, Ni_6_@CuFe-LDH composites with varying Cu–Fe ratios were synthesized and tested for NO_3_RR. All catalysts exhibit a higher current density after adding NO_3_^–^ at the same potential in LSV curves. Figure S28 reveals that the current density gradually decreases with increasing Fe content. Correspondingly, the formation rate decreases as the Fe proportion rises, following the order: Ni_6_@Cu_3_Fe_1_-LDH > Ni_6_@Cu_2_Fe_2_-LDH > Ni_6_@Cu_1_Fe_3_-LDH. However, the FEs of these Ni_6_@CuFe-LDH composites are similar. The Ni_6_@Cu_3_Fe_1_-LDH composites showed the largest C_dl_ values (Figure S18), implying that the copper population in CuFe-LDH is positively proportional to the NO_3_^–^ conversion to NO_2_^–^ intermediate species, which can be further converted to NH_3_ (final product) with the aid of anchored Ni_6_ clusters.

To further elucidate the performance variations of NO_3_RR across different catalysts, we conducted NO_2_RR tests to gain information on the intermediate NO_2_^–^. As depicted in the LSV curves (Figure 3a), all catalysts exhibited significantly higher current density in the electrolyte containing NO_2_^–^ compared to the blank electrolyte at identical potentials, confirming the enhanced activity toward NO_2_^–^ reduction. As illustrated in Figure 3b and 3c, Ni_6_@CuFe-LDH demonstrated enhanced NH_3_ yield and FE, outperforming CuFe-LDH. This performance discrepancy suggests that CuFe-LDH may experience intermediate accumulation (e.g., *NO) during the NO_2_^–^ reduction, leading to sluggish reaction kinetics and the formation of byproducts.^46^ The exceptional NO_2_RR activity of Ni_6_@CuFe-LDH further corroborates its robust NO_3_RR performance and validates the rationality of its structural design. All catalysts achieved FEs exceeding 90% in NO_2_RR, indicating a high selectivity toward NH_3_ production from NO_2_^–^. Specifically, Ni_6_@CuFe-LDH exhibited FE ≈ 98.9% at −0.5 V vs RHE for NO_2_RR, consistent with its NO_3_RR performance. Then we investigated the performance of electrocatalytic NH_3_ synthesis at different NO_3_^–^ concentrations ranging from 1 mM to 100 mM for potential applications.^47,48^ Figure 3d shows the LSV curves of Ni_6_@CuFe-LDH at different NO_3_^–^ concentrations in 1 M KOH. At a identical potentials, the current density of the catalyst increases with increasing NO_3_^–^ concentration, indicating that the NH_3_ formation rate increases with the increase of NO_3_^–^ concentration. The NO_3_RR performance of Ni_6_@CuFe-LDH at different NO_3_^–^ concentrations is shown in Figure 3e and 3f. The activity of NO_3_RR catalysts is directly influenced by the electrolyte concentration. Interestingly, Ni_6_@CuFe-LDH composites showed similar FE at NO_3_^–^ concentrations of 50 and 100 mM, but significantly decreased FE at NO_3_^–^ concentrations less than 10 mM. For this purpose, we further conducted a systematic Tafel analysis and observed a significant concentration-dependent selective trend (Figure S29). With the decrease of concentration, the Tafel slope increases significantly, which reflects the transformation from the mixed Heyrovsky–Volmer kinetics (high NO_3_^–^ coverage inhibits HER) to the Volmer-dominated process (low NO_3_^–^ allows competitive H* formation). This concentration-dependent kinetic hierarchy emphasizes the inhibition of HER through nitrate-regulated adsorption competition and interfacial water recombination.^49^

**Figure 3:**
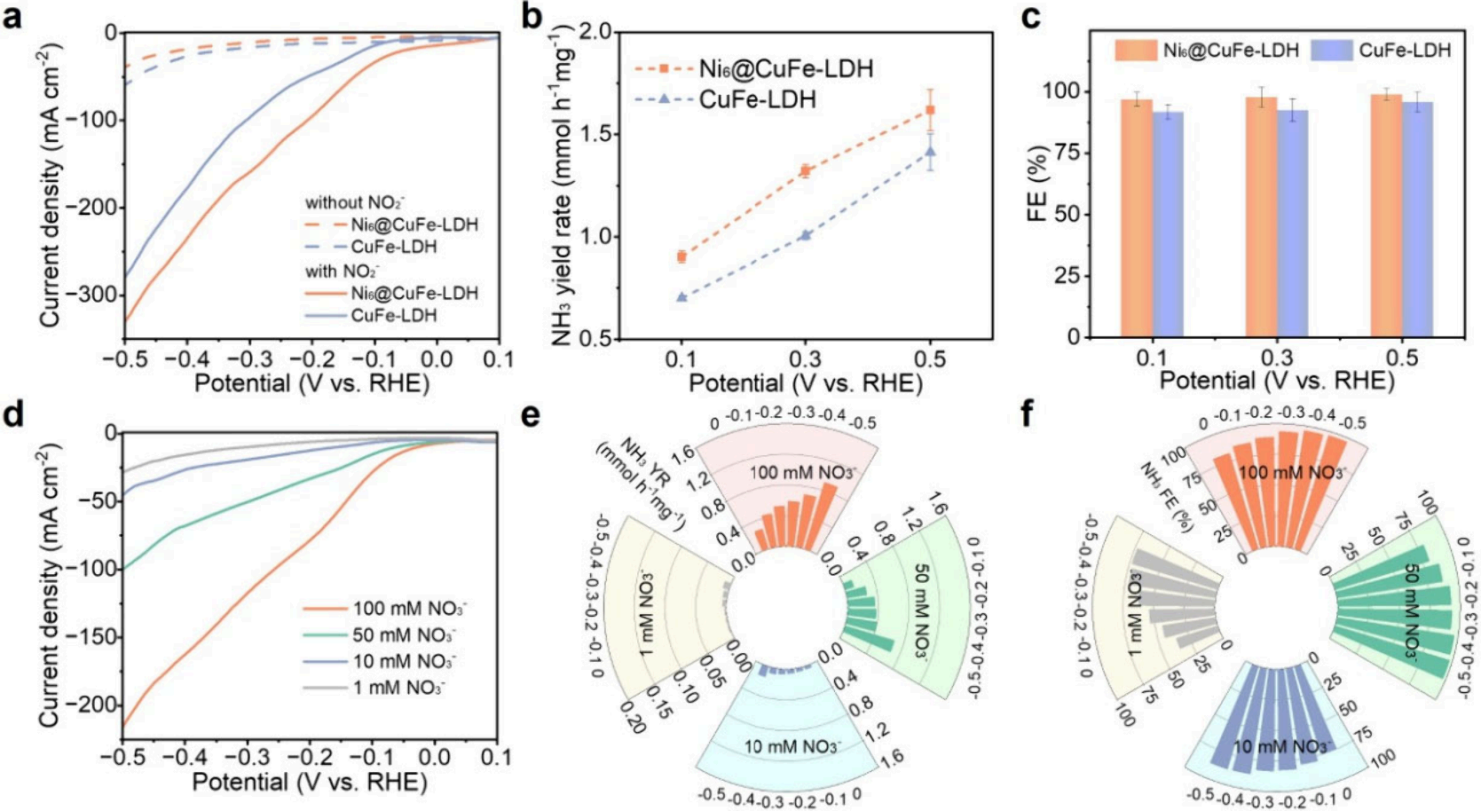
Electrocatalytic performance for NO_2_RR. (a) LSV curves of Ni_6_@CuFe-LDH and CuFe-LDH in 1 M KOH with/without 0.1 M NO_3_^–^ solutions. (b) NH_3_ yield rate and (c) FE of NO_2_RR at various potentials. The electrocatalytic performances of Ni_6_@CuFe-LDH in 1 M KOH electrolyte with different NO_3_^–^ concentrations ranging from 1 mM to 100 mM: (d) LSV curves, (e) NH_3_ formation rate, and (f) FE.

To investigate the catalytic mechanism of NO_3_RR over Ni_6_@CuFe-LDH and CuFe-LDH, in situ Raman spectroscopy and Fourier-transform infrared spectroscopy (FTIR) were employed under the applied potentials ranging from 0 to –0.5 V vs RHE. Raman spectra revealed distinct vibrational modes associated with nitrate reduction intermediates.^50^ As shown in Figure 4a and 4b, the peaks at 1040 and 1372 cm^–1^ were attributed to the symmetric stretching of free aqueous NO_3_^–^ and asymmetric stretching of adsorbed NO_3_^–^ (NO_3_^–^_ad_), respectively.^51^ Notably, the higher intensity ratio of these peaks on Ni_6_@CuFe-LDH compared to CuFe-LDH indicates more nitrate adsorption on the Ni_6_-modified catalyst.^40^ As the potential decreased, Raman bands emerged at 1200 cm^–1^ (adsorbed NO_2_^–^, NO_2_^–^_ad_) and 1344 cm^–1^ (aqueous NO_2_^–^) confirmed the sequential reduction of NO_3_^–^ to NO_2_^–^.^52^ Furthermore, characteristic peaks at 1142 cm^–1^ (N–H bending mode of NH_3_) and 1587 cm^–1^ (symmetric deformation mode of NH_4_^+^) exhibited higher intensities on Ni_6_@CuFe-LDH, demonstrating its advanced capability to promote hydrogenation steps and ammonia generation.^49,53^ Time-dependent Raman spectra at −0.5 V vs RHE showed a gradual decline in NO_3_^–^ peaks and a concurrent rise in NH_3_/NH_4_^+^ signals, highlighting the efficient consumption of nitrate species and progressive formation of final products on Ni_6_@CuFe-LDH (Figure 4c).

**Figure 4:**
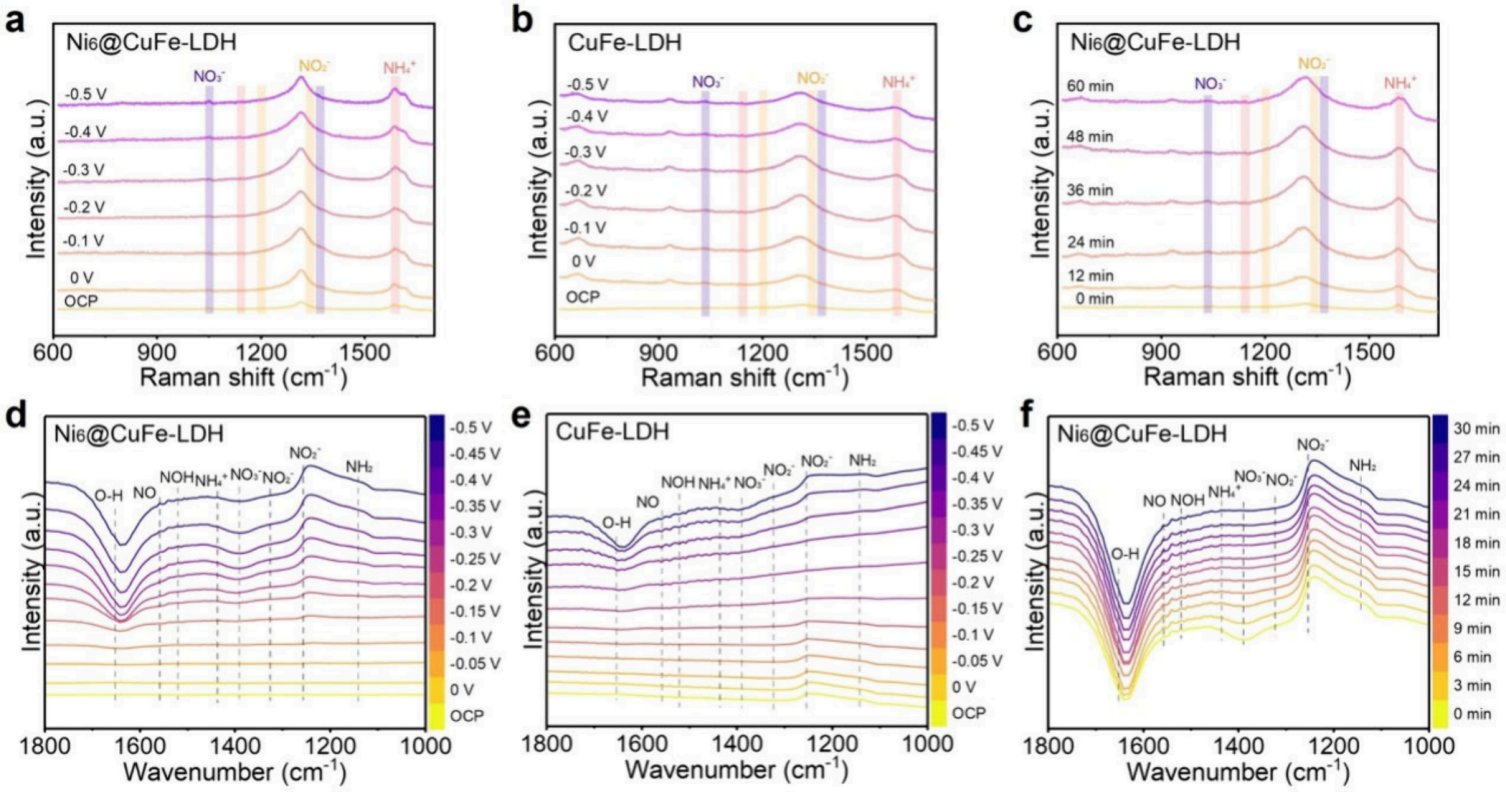
In situ Raman spectra of (a) Ni_6_@CuFe-LDH, (b) CuFe-LDH. (c) Time-dependent in situ Raman spectra of Ni_6_@CuFe-LDH. In situ FTIR spectra of (d) Ni_6_@CuFe-LDH, (e) CuFe-LDH. (f) Time-dependent in situ FTIR spectra of Ni_6_@CuFe-LDH.

In-situ FTIR provides additional mechanistic insights. As shown in Figure 4d, the characteristic absorption band at 1390 cm^–1^ was assigned to the asymmetric stretching vibrations of N–O in NO_3_^–^, signifying the gradual consumption of NO_3_^–^ species.^54^ Concurrently, two distinct peaks appeared at 1325 and 1255 cm^–1^, corresponding to the asymmetric stretching vibrations N–O in NO_2_^–^, which validated the partial reduction of NO_3_^–^ to NO_2_^–^.^55^ Notably, the bending vibrations of surface-adsorbed *NO (1558 cm^–1^) and *NOH (1521 cm^–1^) were also detected, suggesting that the NO_3_RR takes place in the following reaction pathway: NO_3_^–^ → *NO_3_ → *NO_2_ → *NO_2_H→ *NO → *NOH → *N → *NH → *NH_2_ → *NH_3_ → NH_3_.^56^ Furthermore, the peaks at 1142 and 1437 cm^–1^ were attributed to the N–H bending vibrations of *NH_2_ and symmetric deformation vibration of NH_4_^+^, respectively.^57^ Compared to Ni_6_@CuFe-LDH, the in situ FTIR of CuFe-LDH exhibited stronger peaks for NO_2_^–^ intermediates (Figure 4e). This observation implies a constraint in the conversion of NO_2_^–^ on the surface of CuFe-LDH, indicating that the NO_2_^–^ gradually accumulates on catalyst surfaces, leading to slow hydrogenation kinetics on the CuFe-LDH catalyst. Additionally, the O–H bending vibration of surface-adsorbed H_2_O at 1652 cm^–1^ on CuFe-LDH was weaker than that on Ni_6_@CuFe-LDH, highlighting the enhanced water adsorption capability induced by Ni_6_ clusters.^58^ Time-dependent in situ FTIR measurements at –0.5 V vs RHE revealed a progressive increase in the intensity of peaks associated with deoxygenation intermediates (*NO_2_, *NO), hydrogenation intermediates (*NH_2_) and the final product NH_4_^+^ over 30 min (Figure 4f). This kinetic evolution underscores the synergistic role of Ni_6_ clusters and CuFe-LDH in accelerating NO_3_RR activation.

### 3.3 DFT Calculations

To further elucidate the reaction mechanism of NO_3_RR catalyzed by CuFe-LDH and Ni_6_@CuFe-LDH, we performed density functional theory (DFT) calculations. Based on experimental structural characterization, we constructed the optimal adsorption model of each intermediate species on the catalyst surface. To determine the most favorable active sites for intermediate adsorption, we calculated the Gibbs free energy profiles of NO_3_RR on both the Cu and Fe sites of CuFe-LDH (reaction configurations are shown in Figures S30 and S31). The Gibbs free energy profiles for NO_3_RR reveal the reaction pathway: NO_3_^–^ + 6H_2_O + 8e^–^ → NH_3_ + 9OH^–^, which proceeds through deoxygenation steps (*NO_3_^–^ → *NO_2_ → *NO → *N) followed by hydrogenation steps (*N → *NH → *NH_2_ → *NH_3_). As shown in Figure S32, the energy barrier (*E*_a_) for the rate-determining step (RDS) of *NH_3_ desorption on the Fe site (*E*_a_ = 1.41 eV) is slightly lower than that on the Cu site (*E*_a_ = 1.69 eV), which supports the assignment of Fe as the preferred active site in the pristine LDH system. Moreover, we also calculated the free energy profiles for NO_3_RR on the Fe site (originating from CuFe-LDH) and Ni site (originating from the Ni_6_ cluster) of Ni_6_@CuFe-LDH (reaction configurations are shown in Figures S33 and S34). As shown in Figure S35, both the Fe site (*E*_a_ = 0.70 eV) and the Ni site (*E*_a_ = 0.69 eV) exhibit very low activation barriers for the *NO_3_^–^ → *NO_2_ step, which is identified as the new RDS. Notably, the introduction of the Ni_6_ cluster alters the RDS from *NH_3_ desorption to *NO_3_^–^ → *NO_2_. This transition can be attributed to the enhanced entropic compensation provided by the Ni_6_ cluster when NH_3_ transitions from the adsorbed state to the gas phase. The Ni_6_ cluster modulates the adsorption strength of NH_3_, thereby facilitating its desorption and ultimately shifting the RDS. The free energy of CuFe-LDH (Fe site) and Ni_6_@CuFe-LDH (Ni site) is shown in Figure 5a. A similar conclusion was drawn for NO_2_RR using the same computational framework (Figure S36).

**Figure 5:**
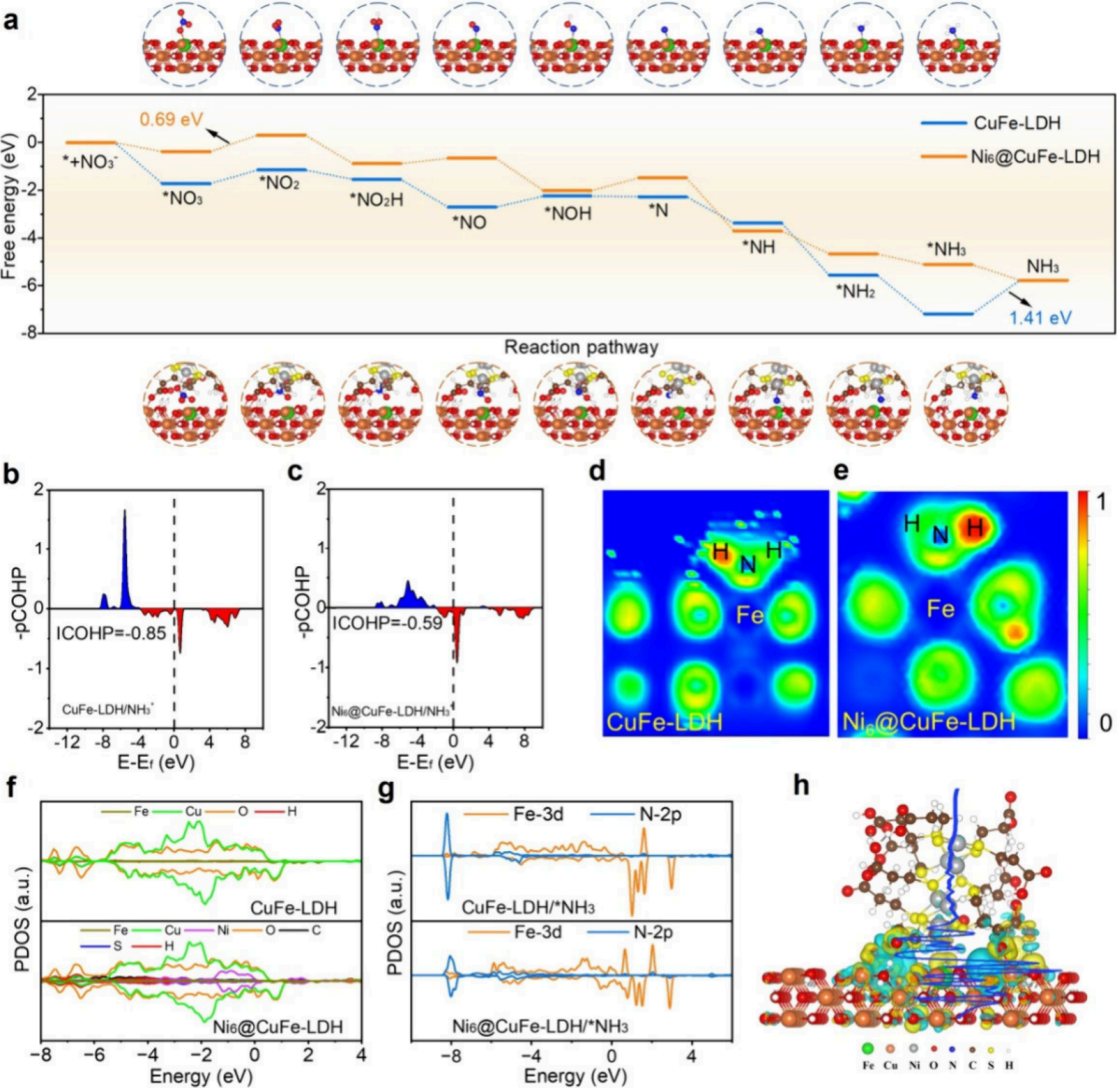
(a) The free energy of CuFe-LDH and Ni_6_@CuFe-LDH. The crystal orbital Hamilton population of (b) CuFe-LDH/*NH_3_ and (c) Ni_6_@CuFe-LDH/*NH_3_. The electron localization function of (d) CuFe-LDH and (e) Ni_6_@CuFe-LDH. (f) The project density of states of CuFe-LDH and Ni_6_@CuFe-LDH. (g) Projected state density of *NH_3_ adsorbed on CuFe-LDH and Ni_6_@CuFe-LDH. (h) Charge density difference and planar-average charge density (blue curve) of Ni_6_@CuFe-LDH. The dashed line represents the Fermi level (isosurface = 0.001 electron/bohr3) and the yellow electron cloud represents accumulation and the cyan electron cloud represents dissipation.

To probe the electronic interactions between *NH_3_ and the catalyst surfaces, we conducted COHP (crystal orbital Hamilton population) analysis, which quantifies the bonding and antibonding characteristics between the active site and the adsorbate. As shown in Figure 5b and 5c, the integrated COHP (ICOHP) value for *NH_3_ on CuFe-LDH is −0.85, while that for *NH_3_ on Ni_6_@CuFe-LDH is −0.59. The more negative ICOHP value for CuFe-LDH indicates a stronger bonding interaction with NH_3_, which explains the unfavorable desorption. In contrast, the incorporation of Ni_6_ clusters into the CuFe-LDH structure weakens this interaction, facilitating *NH_3_ desorption and thereby improving catalytic turnover. Further verify the bonding differences from a real-space electron distribution perspective, we analyzed the electron localization function (ELF) of the two systems (Figure 5d and 5e). The ELF map of CuFe-LDH/*NH_3_ reveals a higher degree of electron localization between the Fe and N atoms, indicating stronger covalent interaction. In contrast, the Ni_6_@CuFe-LDH/*NH_3_ system shows more delocalized electron distribution between Fe and N, further confirming a weaker bonding interaction. These ELF results are in excellent agreement with the COHP and adsorption free energy analyses, collectively demonstrating that the introduction of Ni_6_ clusters reduces the *NH_3_ binding strength, facilitates desorption, and thereby modulates the RDS of the catalytic cycle.

Furthermore, to better correlate these findings with the electronic structure, we calculated the projected density of states (PDOS), as shown in Figure 5f. Near the Fermi level, Ni_6_@CuFe-LDH exhibits a higher density of states, primarily attributed to the contribution of the Ni_6_ cluster, which enhances the electronic conductivity. We further calculated the PDOS after *NH_3_ adsorption, focusing on the Fe 3d and N 2p orbitals (Figure 5g). Although Ni_6_@CuFe-LDH displays a slightly more pronounced d-p orbital overlap near the Fermi level, this does not directly indicate stronger adsorption. Instead, it reflects greater electronic participation, which may promote electron transfer and catalytic turnover. Importantly, the key insight arises from the combination of COHP and PDOS analyses: while the orbital overlap (PDOS) appears stronger in Ni_6_@CuFe-LDH, the net bonding interaction (COHP) is weaker, leading to a more favorable desorption of *NH_3_. Therefore, the change in the RDS is better explained by the electronic modulation of *NH_3_ interaction. This synergistic effect is further confirmed by the charge density difference and planar-average charge density analysis (Figure 5h). To assess the generality of this effect, we extended the analysis to other systems. As shown in Figures S37–S39, the Au_9_@CuFe-LDH system also exhibits weakened *NH_3_ adsorption, resulting in a similar shift in the RDS from *NH_3_ desorption to *NO_3_ → *NO_2_ conversion, consistent with the behavior observed in Ni_6_@CuFe-LDH. Moreover, as illustrated in Figure S40, we analyzed the charge density difference between the metallic cluster and the CuFe-LDH substrate. In both Ni_6_@CuFe-LDH and Au_9_@CuFe-LDH systems, significant interfacial charge redistribution is observed, further supporting the interfacial synergy hypothesis. In addition, considering the competing hydrogen evolution reaction (HER), we calculated the corresponding free energy profiles (Figures S41 and 42). The Ni_6_@CuFe-LDH system displays a larger HER free energy barrier (*E*_a_ = 0.92 eV) compared to that of CuFe-LDH (*E*_a_ = 0.46 eV), indicating its suppressed HER activity and enhanced selectivity toward nitrate reduction^49,59^ In summary, the introduction of metal clusters such as Ni_6_ and Au_9_ modulates the local electronic environment and optimizes intermediate interactions, collectively tuning the reaction pathway, suppressing side reactions, and improving catalytic performance.

## 4 Conclusion

In summary, we have prepared Ni_6_@CuFe-LDH composites by combining Ni_6_(SC_2_H_4_COOH)_12_ clusters and CuFe-LDH nanosheets via electrostatic interactions. Such Ni_6_@CuFe-LDH catalysts presented a promising NH_3_ productivity of 0.91 mmol mg^–1^ h^–1^ with a high faradaic efficiency of ∼97% in electrochemical NO_3_RR to ammonia. Although the NH_3_ productivity over Ni_6_@CuFe-LDH is similar to that of CuFe-LDH, the NH_3_ faradaic efficiency largely surpasses that of CuFe-LDH (∼73% FE). The mechanism involves the synergism between Ni_6_ and CuFe-LDH to promote the conversion of nitrite intermediates to ammonia as the deeply hydrogenated product. This mechanism is confirmed by the control experiments of NO_2_RR and further computations. The *NO_3_^–^ → *NO_2_ step is determined to be the RDS for the Ni_6_-based catalysts, differing from the RDS for pristine CuFe-LDH (the RDS being the desorption of *NH_3_ species). In all, these efforts serve as an attempt to push forward the research frontier in metal clusters for electrochemical investigations at the precise atomic-level.

## Supplementary Material



## Notes

Additional experiments and catalyst characterization data, STEM images with EDS, XPS, UV–vis, and DFT study (PDF)
