# Supplementary material for: Tiara Ni Clusters for Electrocatalytic Nitrate Reduction to Ammonia with 97% Faradaic Efficiency
Source: J Am Chem Soc. 2025 Jun 20;147(26):22785–95. doi: 10.1021/jacs.5c04950 (PMC12232310; doi:10.1021/jacs.5c04950)
Supplement: Supplementary file 1 [file ja5c04950_si_001.pdf]

Supporting Information

Tiara Ni Clusters for Electrocatalytic Nitrate Reduction to Ammonia with 97% Faradaic Efficiency

Xinrui Gu,[‡,$#] Jingjing Zhang,[†,‡#] Song Guo,[‡] Yifei Zhang,[‡] Liangliang Xu,[Δ*] Rongchao Jin,[δ*] Gao Li[†,‡*]

[†] School of Chemistry and Chemical Engineering, Inner Mongolia Normal University, Hohhot 010018, China

[‡] State Key Laboratory of Catalysis, Dalian Institute of Chemical Physics, Chinese Academy of Sciences, Dalian 116023, China;

[Δ] Department of Chemistry, University of Puerto Rico, Rio Piedras, San Juan, PR 00931, United States;

[δ] Department of Chemistry, Carnegie Mellon University, Pittsburgh, PA 15213, United States;

[$] University of Chinese Academy of Sciences, Beijing 100049, China.

Experimental Section

Chemicals

Iron nitrate nonahydrate ($Fe(NO_3)_3·9H_2O$, 98.5%), cupric nitrate trihydrate ($Cu(NO_3)_2·3H_2O$, 99%), ammonium chloride (NH_4Cl, 99.5%), sodium hydroxide (NaOH, 96%), anhydrous sodium carbonate (Na_2CO_3, 99.8%), potassium nitrate (KNO_3, 99%), and sodium citrate ($Na_3C_6H_5O_7$, 99%) were purchased from Hushi. Nickel acetate ($Ni(CH_3COO)_2·4H_2O$, 98%) and phosphoric acid (H_3PO_4, 85 wt%) were obtained from Kermel. Mercaptopropionic acid ($C_3H_6O_2S$, 98%), Nafion solution (5 wt%), Vulcan XC-72R carbon powder, potassium nitrite (KNO_2, 97%), salicylic acid ($C_7H_6O_3$, 99%), sodium nitroferricyanide dihydrate ($Na_2Fe(CN)_5NO·2H_2O$, 99%), sodium hypochlorite solution ($NaClO_3·5H_2O$, 40%), sulfanilamide ($C_6H_8N_2O_2S$, 99%) and N-(1-Naphthyl) ethylenediamine dihydrochloride ($C_{12}H_{14}N_2·2HCl$, 99%) were obtained from Aladdin. Potassium hydroxide (KOH, 90%) was obtained from General Reagent. Deuterium Oxide (D_2O) was purchased from damas-beta. Ultrapure water (>18.2 MΩ cm, Sartorius Arium Comfort I system) was used to prepare solutions and wash glass in all experiments.

Characterization

Ultraviolet−visible (UV–vis) absorption spectroscopy was carried out on a Shimadzu UV-1800 spectrophotometer. Transmission electron microscopy (TEM) and high-angle annular dark-field scanning TEM (HAADF-STEM) were conducted on a JEM-F200 (JEOL Ltd.). The powder X-ray diffraction (XRD) patterns were obtained on a Rigaku X-ray powder diffractometer (SmartLab, Rigaku Corporation), using Cu Kα radiation at 40 kV and 30 mA. High resolution X-ray photoelectron spectroscopy (XPS) analyses were performed on a Theta Probe system (base pressure: ~10^{-8} Pa), equipped with a Phoibos 100 hemispherical analyzer and XR 50 X-ray source (SPECS GmbH) operating in the constant pass energy mode at 50 eV. All spectra were corrected using the C 1s signal (284.8 eV) with CasaXPS software. X-ray absorption spectroscopy (XAS) measurements were performed on a laboratory device (easyXAFS300, easyXAFS LLC).

Zeta potential measurements were carried out on Nano Brook 90Plus PALS. ^1H nuclear magnetic resonance (NMR) was performed on AVANCE III 400 MHz (Bruker). In situ Raman experiments were conducted using a microscopic laser confocal Raman spectrometer (inVia, Renishaw) equipped with a 785 nm excitation laser. In situ Fourier-transform infrared spectroscopy (FTIR) measurements were performed on VERTEX 80V spectrometer (Bruker) equipped with a germanium selenide crystal substrate.

Synthesis of $Ni_6(SC_2H_4COOH)_{12}$ clusters

Typically, mercaptopropionic acid (MPA) and one equivalent of NaOH were dissolved in water and stirred. Half equivalent of $Ni(OAc)_2\cdot 6H_2O$ aqueous solution was added into the above solution. After one hour, the clusters in the supernatant were separated by centrifugation and stored for preparation of Ni_6@CuFe-LDH composites.

Determination of products

The concentration of NH_3 in the reaction solution was determined using colorimetry with the indophenol blue method. Specifically, an appropriate amount of electrolyte was removed from the reaction cell, then 2 mL of a 1 M NaOH solution containing 5wt.% salicylic acid and 5 wt.% sodium citrate was added, followed by the addition of 1mL of 0.1 M NaClO and 0.2 mL of 1 wt.% sodium nitroprusside dihydrate aqueous solution. After standing at room temperature for 2 h in the dark, the UV-vis absorption spectrum was measured. The concentration of NH_3 was determined based on the absorbance at 655 nm. The concentration absorbance curves were calibrated using standard ammonia chloride solutions (Fig. S3).

The concentration of NO_2^- in the reaction solution was determined using the Griess test. The Griess reagent was prepared by dissolving 0.02 g N-(1-Naphthyl) ethylenediamine dihydrochloride, 0.4 g sulfonamide, and 1 mL phosphoric acid (85%) in 5 mL ultrapure water. To determine the concentration of NO_2^-, 0.1 mL of the tested electrolyte was mixed with 0.1 mL of Griess reagent to react at room temperature for 20 min under dark conditions. Then, the electrolyte was diluted to the measurable concentration range. The concentration of NO_2^- was determined by absorbance around 540 nm. Calibration of concentration-absorbance curves was performed using standard potassium nitrite solutions (Figure S3).

Calculation of the reaction rate and the Faradaic efficiency of NH_3 and NO_2^-

The NH_3 formation rate (mmol h^{-1} mg^{-1}) was calculated by the following equation: $\mathrm{YR}\ (\mathrm{NH_3}) = \frac{c_{\mathrm{NH_3}} \times V}{17 \times t \times m_{cat}}$

The Faradaic efficiency (FE) of NH_3 can be calculated by the following equation: $\mathrm{FE}\ (\mathrm{NH_3}) = \frac{8 \times F \times c_{\mathrm{NH_3}} \times V \times 10^{-6}}{17 \times Q} \times 100\%$

The NO_2^- yield rate (mmol h^{-1} mg^{-1}) was calculated by the following equation: $\mathrm{YR}\ (\mathrm{NO_2^-}) = \frac{c_{\mathrm{NO_2^-}} \times V}{46 \times t \times m_{cat}}$

The Faradaic efficiency (FE) of NO_2^- was calculated by the following equation: $\mathrm{FE}\ (\mathrm{NO_2^-}) = \frac{2 \times F \times c_{\mathrm{NO_2^-}} \times V \times 10^{-6}}{46 \times Q} \times 100\%$

Where, c_{NH3} is the detected NH_3 concentration of electrolyte, c_{NO2-} is the detected NO_2^- concentration of electrolyte, V is the volume of the electrolyte (40 ml), t is the electrolysis time (1 h), m_{cat} is the mass of catalyst (0.15 mg), F is the Faraday constant (96500 C mol^{-1}), and Q is the charge passing through the electrode, which is the integral of I-t curve.

Isotope labeling experiment

The isotope labeling experiment was conducted with 98% KNO_3 as the nitrogen source. Under the same reaction conditions (1 M KOH and 0.1 M NO_3^-, at −0.5 V vs. RHE), after electrolysis for 2 hours, the liquid phase products were acidified to pH=3 with 0.5M H_2SO_4, and then deuterium oxide (D_2O) was sealed in the internal standard tubes for ^1H nuclear magnetic resonance (NMR) analysis to distinguish $^{14}NH_4^+$ and $^{15}NH_4^+$ products.

TOF calculation

The values of turnover frequency (TOF) are calculated via the following formula:

$$\mathrm{TOF} = \frac{\mathrm{j} \times \mathrm{A}/(\mathrm{z} \times \mathrm{F})}{\mathrm{n}_{\text{active sites}}}$$

where, j is the measured current density ($A \cdot cm^{-2}$) at −0.5 V vs. RHE, A is the electrode area (cm^{-2}), z is the electron transfer number of the target reaction (For NO_3RR, z=8), F is the Faraday constant (96500 C mol^{-1}).

$$\mathrm{n}_{\text{active sites}} = \frac{\mathrm{m} \times \mathrm{wt\%}_{\text{metal}}}{100 \times \mathrm{M}}$$

The number of active sites in catalyst was calculated from the total mass of Ni/Fe on the electrode and the atomic weight of Ni/Fe, if all Ni/Fe atoms contributed to the reaction process. Where, m is the catalyst loading mass (g), $\mathrm{wt\%}_{\text{metal}}$ is the mass percentage of the metal component (%), M is the molar mass of the metal ($g \cdot mol^{-1}$).

In situ Raman experiment

In situ Raman experiments were conducted using a microscopic laser confocal Raman spectrometer (inVia, Renishaw) equipped with a 785 nm excitation laser. Raman frequency calibration was performed with a monocrystalline silicon sample before each experiment. In situ electrochemical Raman measurements were carried out in a 3H gas-diffusion Raman cell (Gaoss Union C031-3), separated by a proton exchange membrane (Nafion 117). The applied potential was controlled by a CHI 760E workstation. The catalyst ink was dropped on carbon paper, ensuring the sample plane was perpendicular to the incident laser. A Hg/HgO electrode (filled with 1M KOH) and a graphite rod were used as reference and counter electrodes, respectively. The electrolyte was a mixed solution of 1 M KOH and 0.1 M KNO_3.

In situ FTIR experiment

In situ Fourier-transform infrared spectroscopy (FTIR) measurements were performed on VERTEX 80V spectrometer (Bruker) equipped with a germanium selenide crystal substrate. The applied potential was regulated by a CHI 760E workstation. For the in situ FTIR testing, the catalyst was deposited onto a carbon paper substrate (1×1 cm^2). A Hg/HgO electrode and a platinum wire were served as reference and counter electrodes, respectively. The electrolyte consisted of a mixed solution of 1 M KOH and 0.1 M KNO_3.

Density functional theory calculations

All calculations were performed using the Vienna Ab initio Simulation Package (VASP),[1] with structural optimizations

based on density functional theory (DFT). The exchange–correlation energy was modeled using the generalized gradient approximation (GGA) as proposed by Perdew–Burke–Ernzerhof (PBE).[2] The valence electronic states were expanded using plane waves, while core–valence interactions were described by the projector augmented wave (PAW) method,[3] with a cutoff kinetic energy of 400 eV. The bottom two layers of the model was fixed, while all other atoms, were fully relaxed during geometry optimization. The Brillouin zone was sampled using a Gamma-centered 1×1×1 and 2×2×1 k-point grid for geometry optimization and electronic property calculations, respectively,[4] chosen to accommodate the large structural model comprising approximately 300 atoms. Structural relaxations were carried out using the conjugate-gradient algorithm until the Hellmann–Feynman forces and energy per atom converged to within 0.05 eV $Å^{-1}$ and 10^{-5} eV, respectively. To mitigate the effects of periodic boundary conditions (PBC), a vacuum layer of 15 Å was introduced along the z-direction to minimize interactions between periodic images. Additionally, van der Waals (vdW) interactions were accounted for using Grimme's DFT-D3 scheme with dispersion correction.[5] Spin polarization was included in all calculations to ensure accurate electronic structure descriptions.

The NO_3RR pathways are depicted as follows:

(1) $* + NO_3^- \rightarrow *NO_3 + e^-$	(2) $*NO_3 + 2H^+ + 2e^- \rightarrow *NO_2 + H_2O$	(3) $*NO_2 + H^+ + e^- \rightarrow *NO_2H$
(4) $*NO_2H + H^+ + e^- \rightarrow *NO + H_2O$	(5) $*NO + H^+ + e^- \rightarrow *NOH$	(6) $*NOH + H^+ + e^- \rightarrow *N + H_2O$
(7) $*N + H^+ + e^- \rightarrow *NH$	(8) $*NH + H^+ + e^- \rightarrow *NH_2$	(9) $*NH_2 + H^+ + e^- \rightarrow *NH_3$
(10) $*NH_3 \rightarrow NH_3 + *$	where, * represents the active site.	

For each reaction, the Gibbs free energy was calculated via the following equation: $\Delta G = E_{DFT} + ZPE - \Delta TS$, where ΔG represents Gibbs free energy, E_{DFT} is the energy obtained by DFT calculations, ZPE and ΔTS are zero-point energy correction and entropy correction, respectively.

Supporting Figures and Tables:

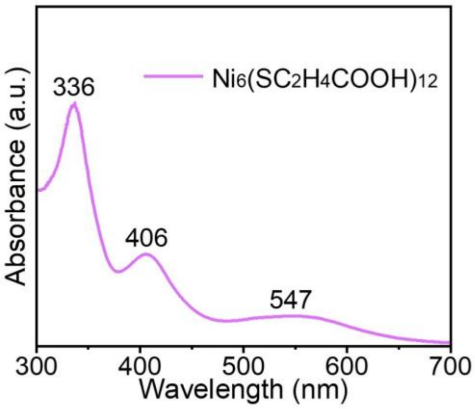

Figure S1. UV-vis spectrum of free $Ni_6(SC_2H_4COOH)_{12}$ clusters in an aqueous solution.

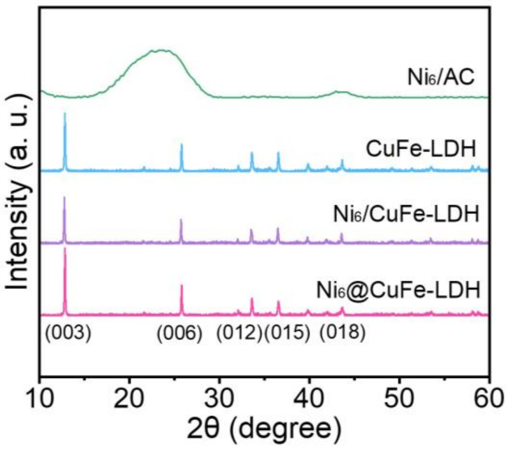

Figure S2.XRD patterns of Ni_6/AC, CuFe-LDH, Ni_6/CuFe-LDH and Ni_6@CuFe-LDH.

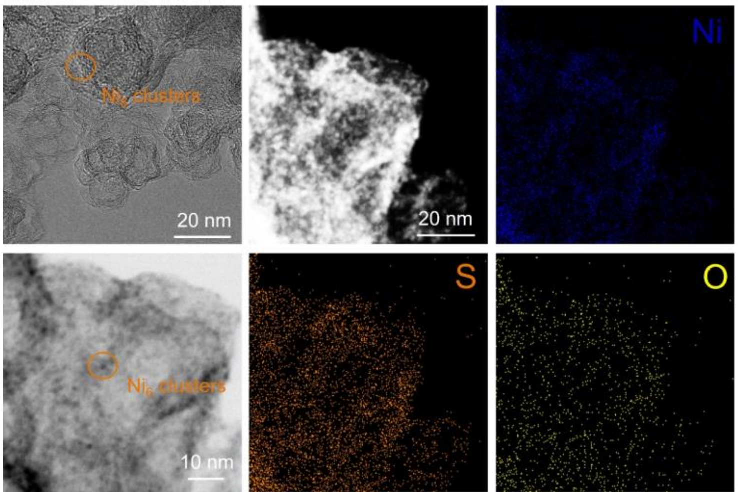

Figure S3. TEM and HAADF-STEM image along with EDX mapping images of nickel (Ni), sulfur (S) and oxygen (O) of Ni_6/AC.

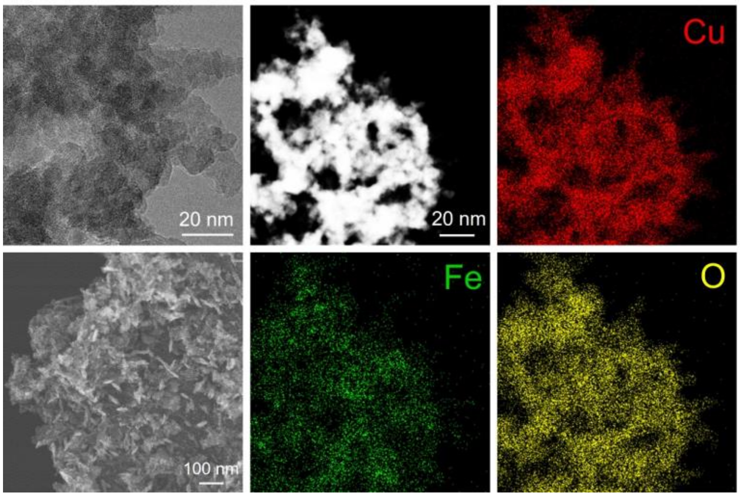

Figure S4. TEM and HAADF-STEM image along with EDX mapping images of copper (Cu), iron (Fe), and oxygen (O) of CuFe-LDH.

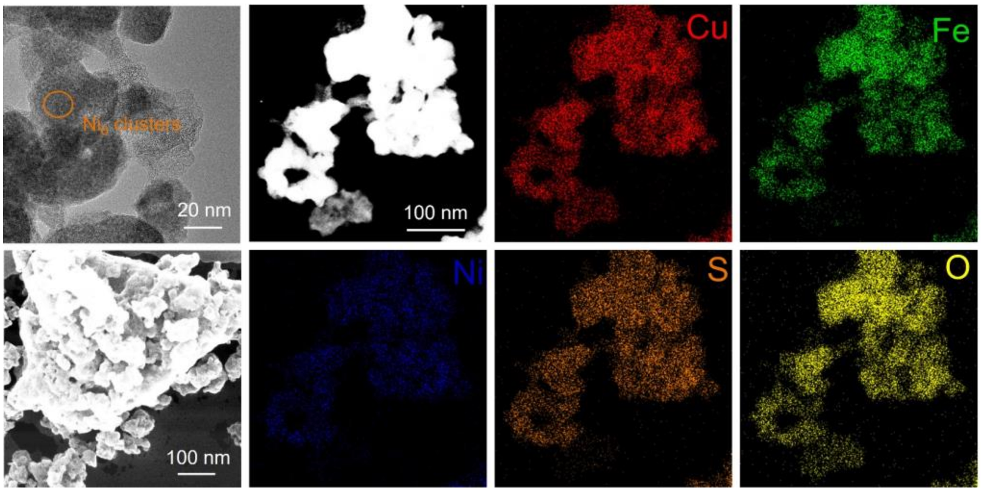

Figure S5. TEM and HAADF-STEM image along with EDX mapping images of copper (Cu), iron (Fe), nickel (Ni), sulfur (S) and oxygen (O) of Ni_6/CuFe-LDH.

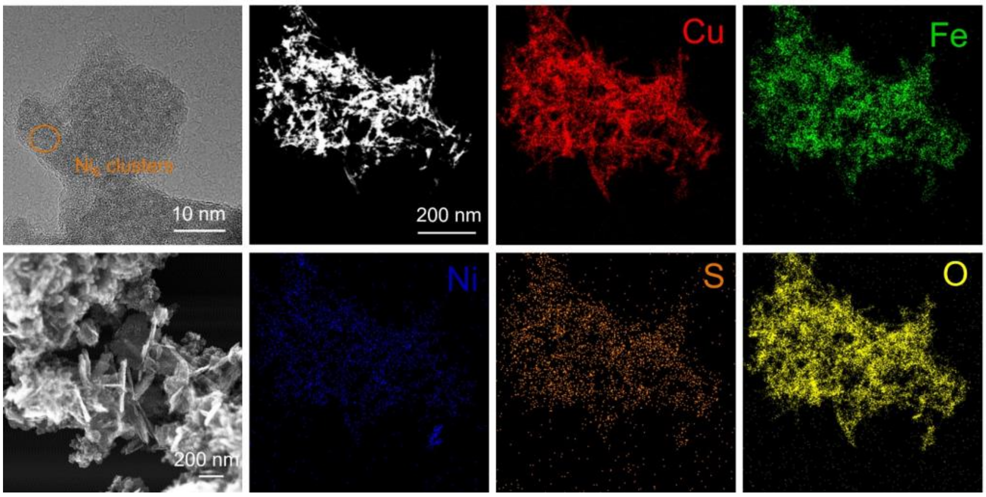

Figure S6. TEM and HAADF-STEM image along with EDX mapping images of copper (Cu), iron (Fe), nickel (Ni), sulfur (S) and oxygen (O) of Ni_6@CuFe-LDH.

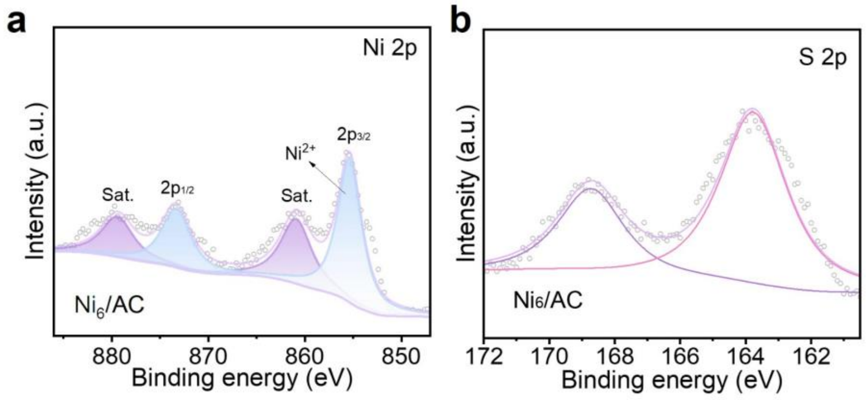

Figure S7. High resolution Ni 2p and S 2p XPS spectra of Ni_6/AC.

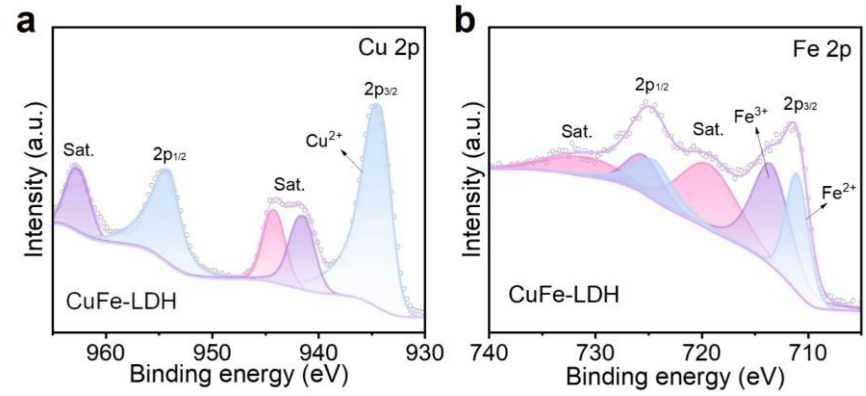

Figure S8 High resolution Cu 2p and Fe 2p XPS spectra of CuFe-LDH.

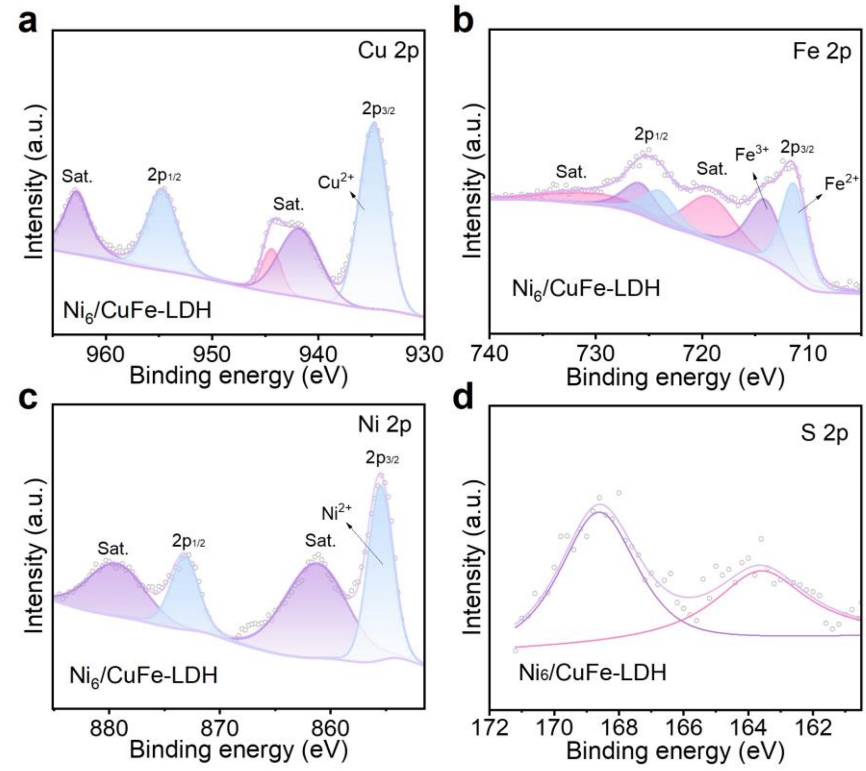

Figure S9 High resolution Cu 2p, Fe 2p, Ni 2p and S 2p XPS spectra of Ni_6/CuFe-LDH.

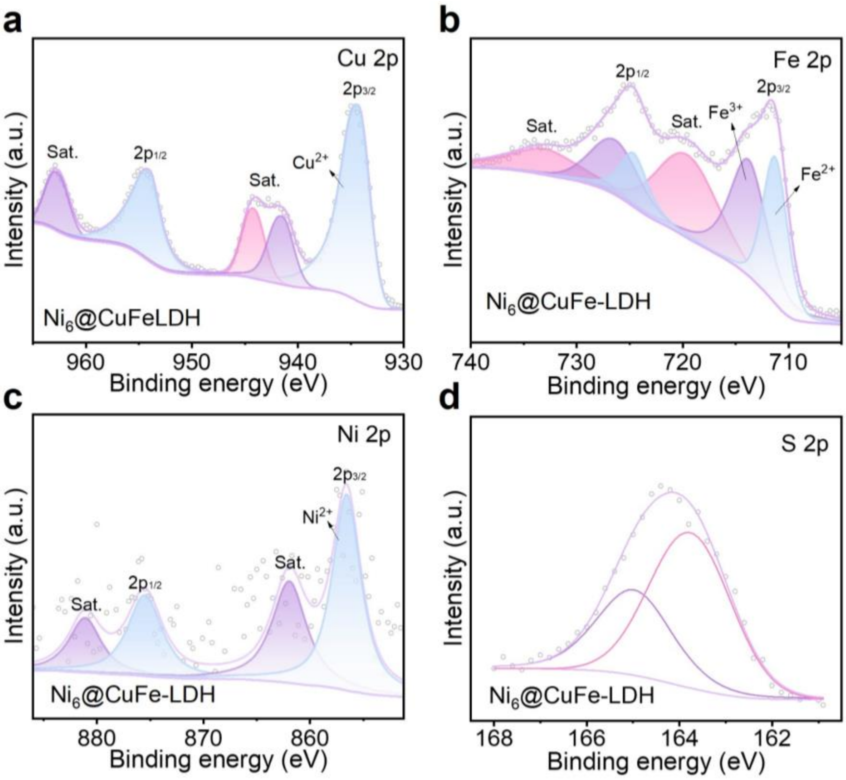

Figure S10 High resolution Cu 2p, Fe 2p, Ni 2p and S 2p XPS spectra of Ni_6@CuFe-LDH.

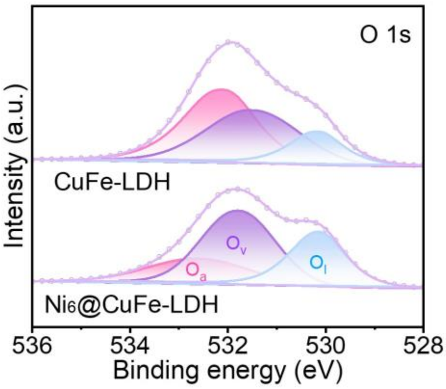

Figure S11 High resolution O 1s spectra of CuFe-LDH and Ni_6@CuFe-LDH.

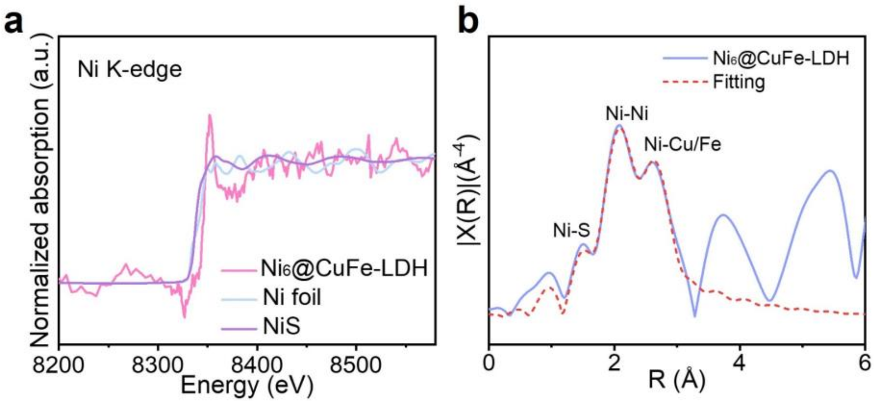

Figure S12. XANES spectra and EXAFS fitting curves of Ni K-edge of Ni_6@CuFe-LDH.

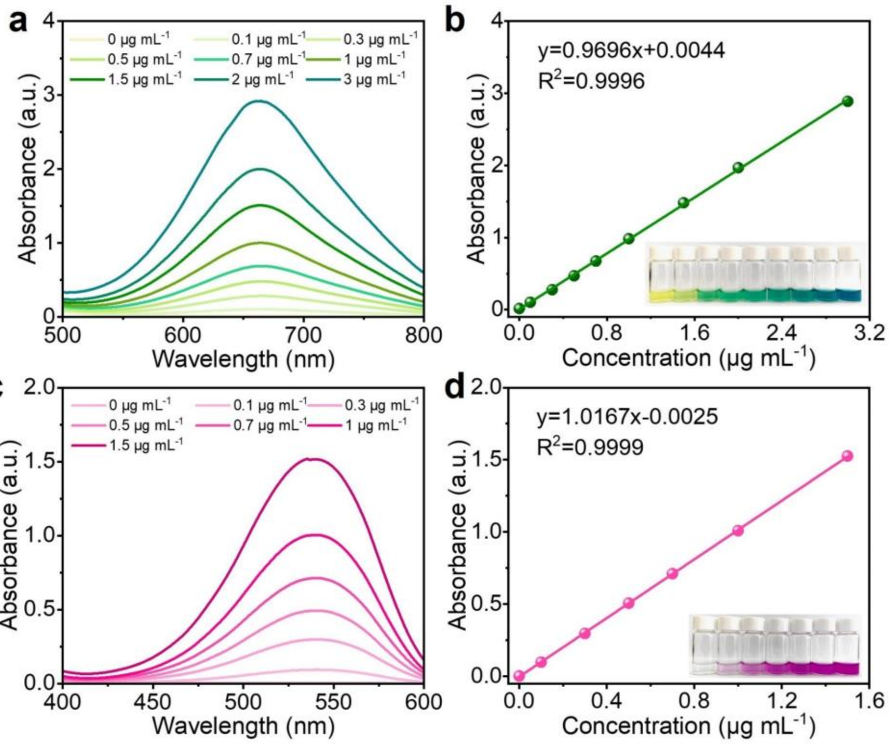

Figure S13. Standard curves for the quantification of NH_3 and NO_2^- by the colorimetric method. (a) UV-vis absorption spectra and (b) standard curves of NH_3 at different concentrations. (c) UV-vis absorption spectra and (d) standard curves of NO_2^- at different concentrations.

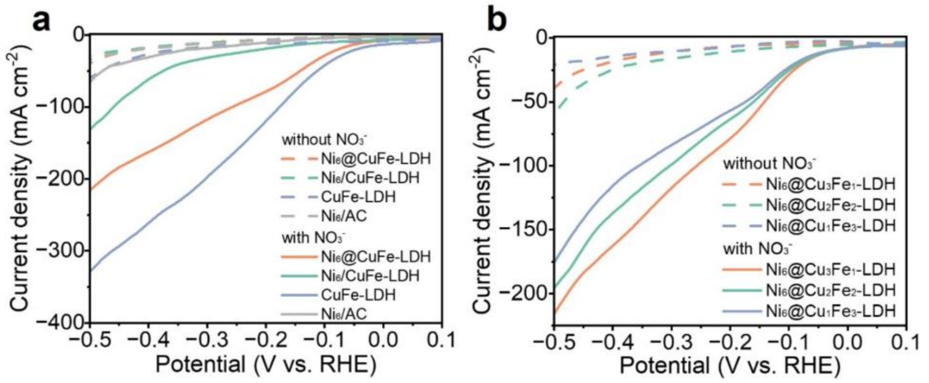

Figure S14. LSV curves in 1 M KOH with and without 0.1 M NO_3^-.

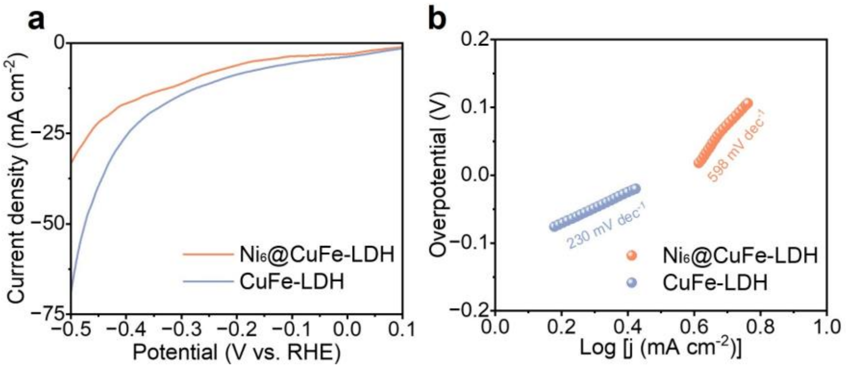

Figure S15. HER performance of Ni_6@CuFe-LDH and CuFe-LDH in 1M KOH. (a) HER LSV curves. (b) Tafel plots.

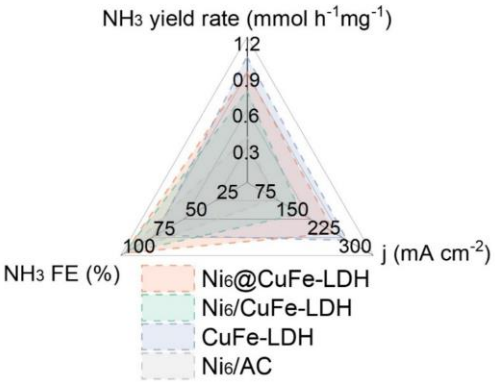

Figure S16. Comparison of the performance of the samples.

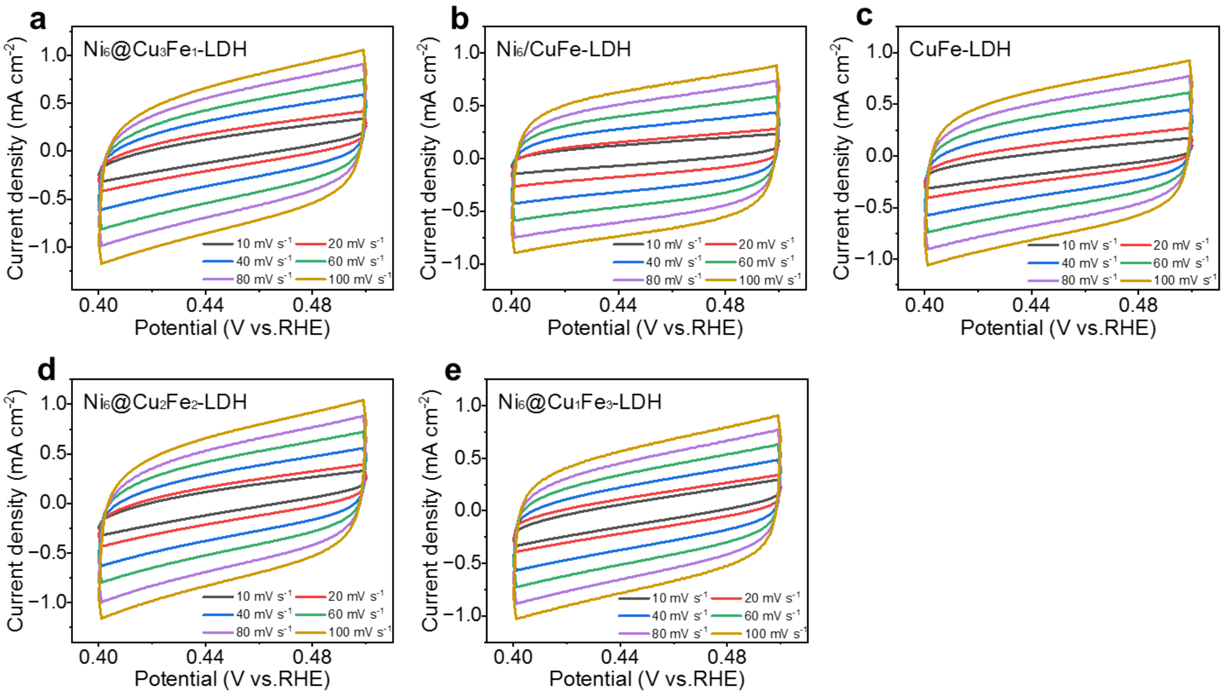

Figure S17. CV curves of the samples at scan rates ranging from 10 to 100 mV s^{-1}.

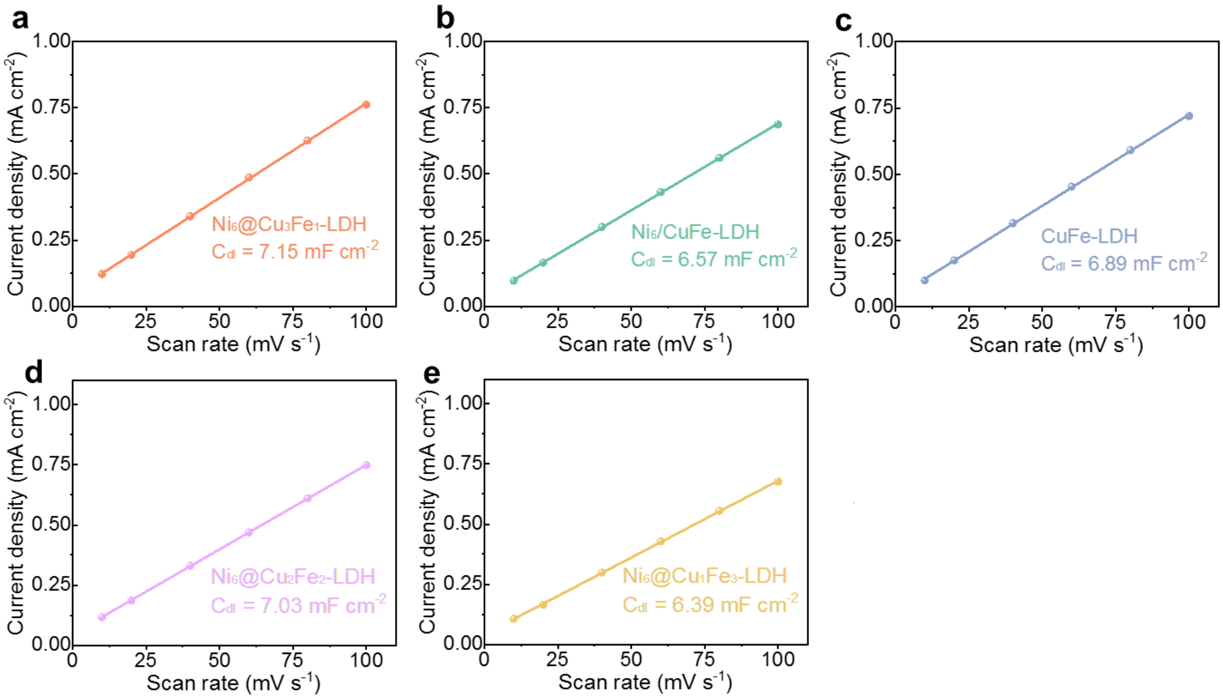

Figure S18. Charging current density differences plotted versus the different scan rates of samples.

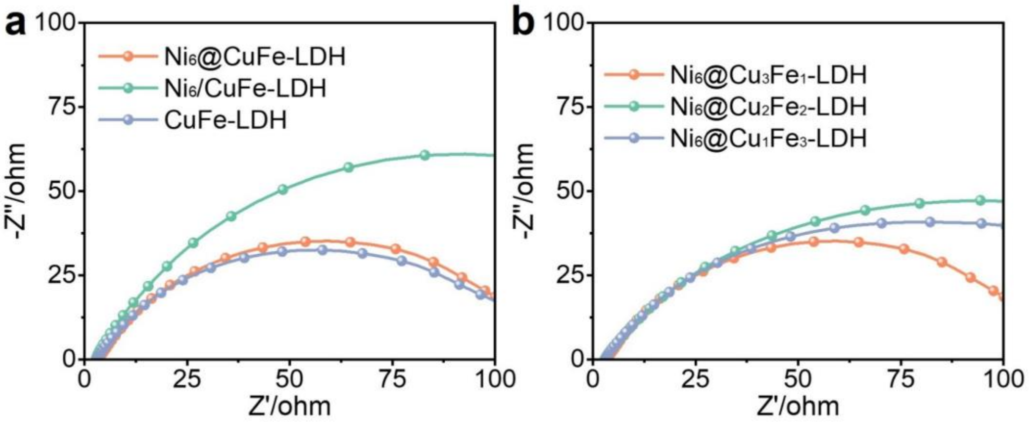

Figure S19. EIS Nyquist plots of the samples.

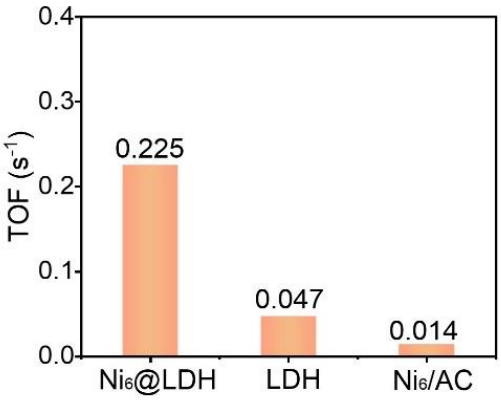

Figure S20. The turnover frequency (TOF) of Ni_6@CuFe-LDH, CuFe-LDH, and Ni_6/AC for converting NO_3^- to NH_3.

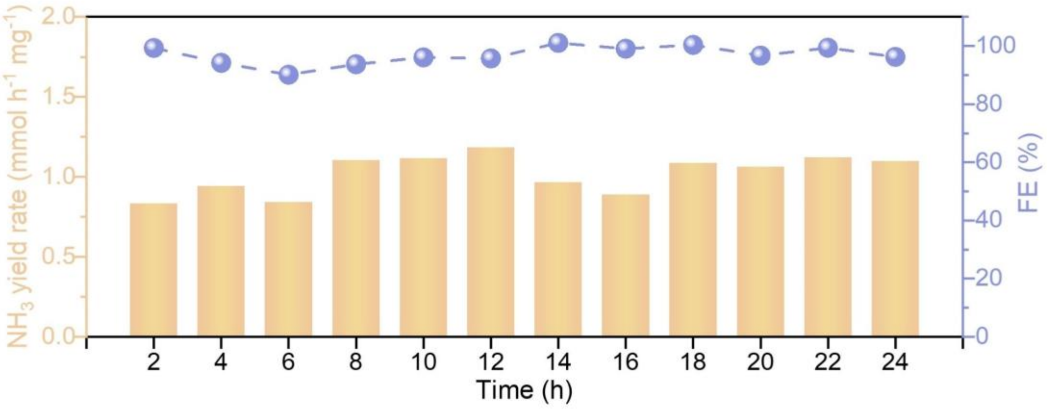

Figure S21. Long-term chronoamperometry tests at -0.5V (vs, RHE).

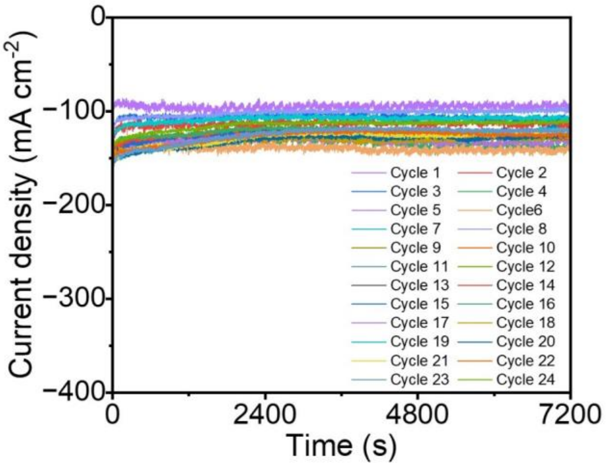

Figure S22. The I-t curves of Ni_6@CuFe-LDH over 24 cycles.

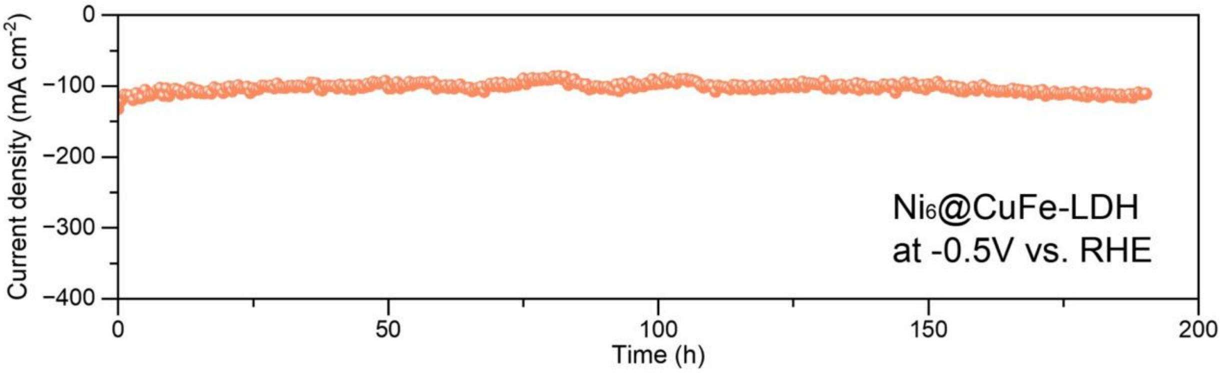

Figure S23. The durability test of Ni_6@CuFe-LDH at -0.5 V (vs. RHE) for ~190h.

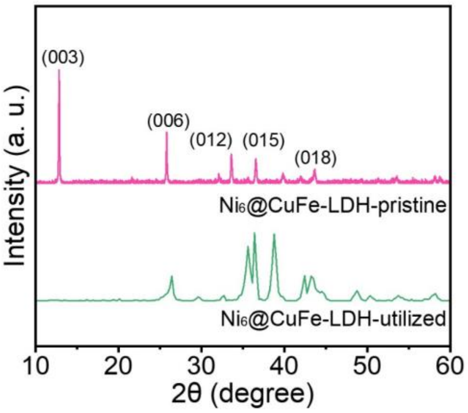

Figure S24. XRD patterns of pristine and used Ni_6@CuFe-LDH.

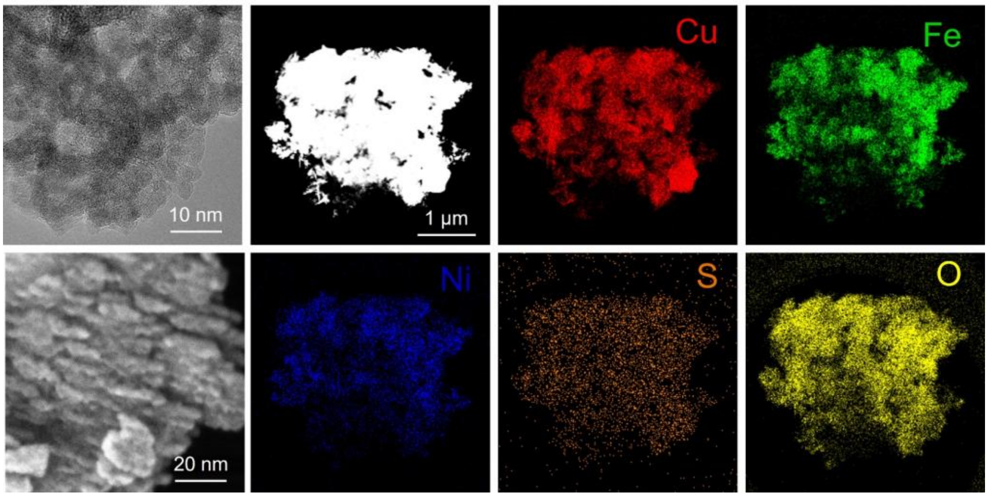

Figure S25. TEM and HAADF-STEM image along with EDX mapping images of copper (Cu), iron (Fe), nickel (Ni), sulfur (S) and oxygen (O) of pristine and used Ni_6@CuFe-LDH.

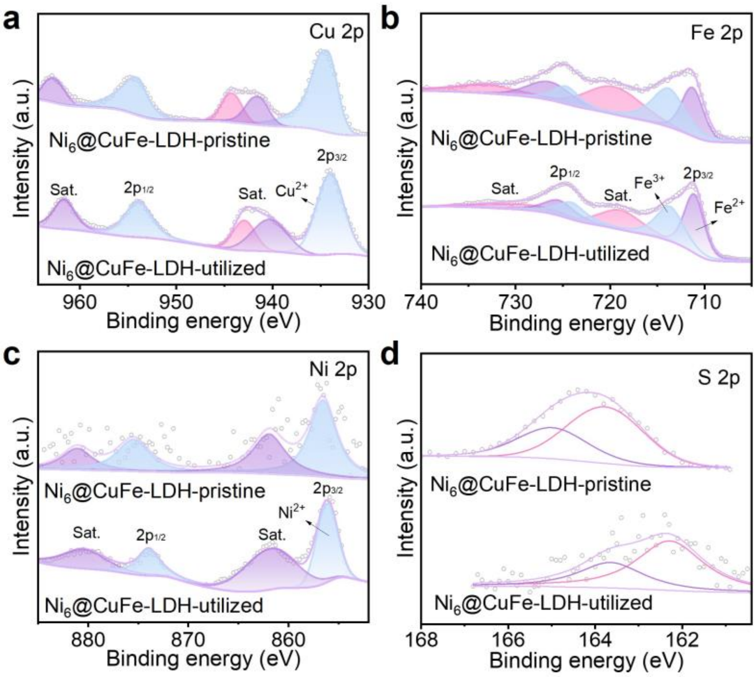

Figure S26. High resolution Cu 2p, Fe 2p, Ni 2p and S 2p XPS spectra of pristine and used Ni_6@CuFe-LDH.

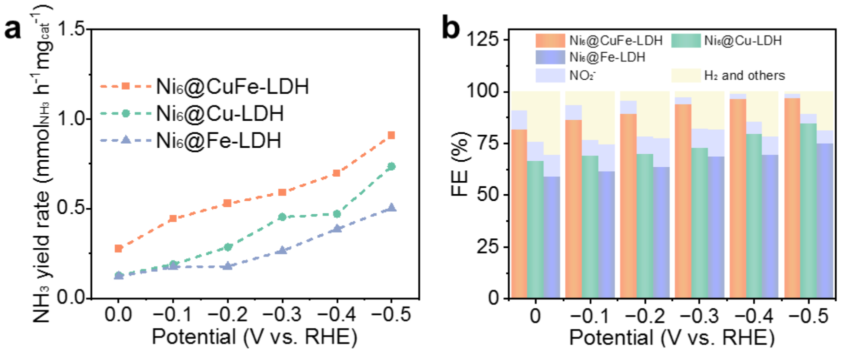

Figure S27. Electrocatalytic performance of NO_3RR on Ni_6@CuFe-LDH, Ni_6@Cu-LDH and Ni_6@Fe-LDH in 1 M KOH with 0.1 M NO_3^-. (a) NH_3 yield rate and (b) FE of NO_3RR at various potentials.

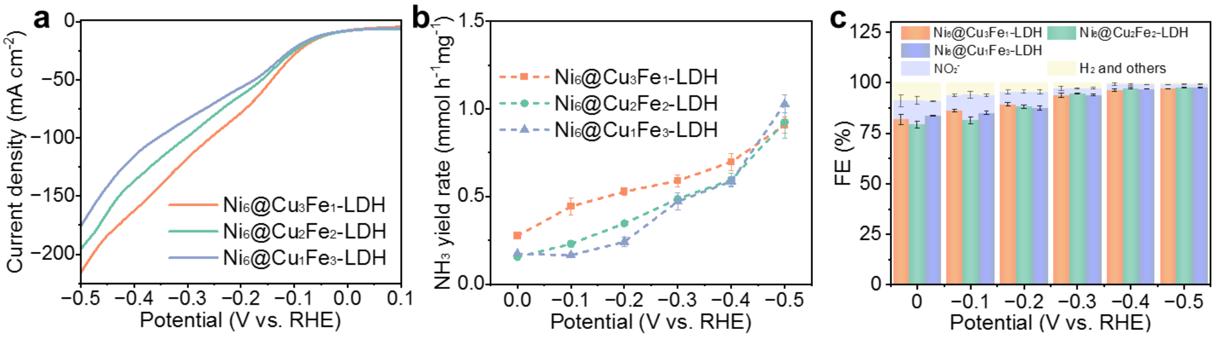

Figure S28. Electrocatalytic performance for NO_3RR. (a) LSV curves of Ni_6@CuFe-LDH with different Cu/Fe ratio in 1 M KOH with 0.1 M NO_3^-. (b) NH_3 yield rate and (c) FE of NO_3RR at various potentials.

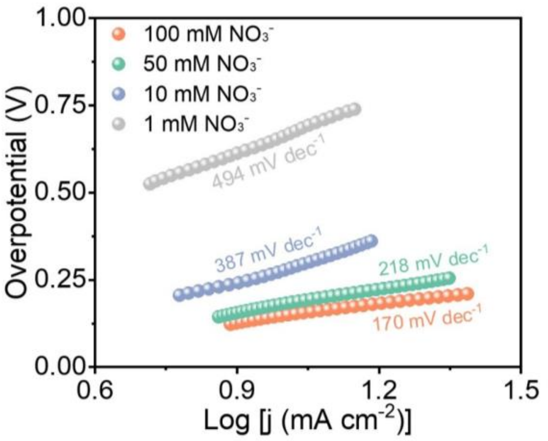

Figure S29. Tafel plots of Ni_6@CuFe-LDH in 1 M KOH electrolyte with different NO_3^- concentrations ranging from 1 mM to 100 mM.

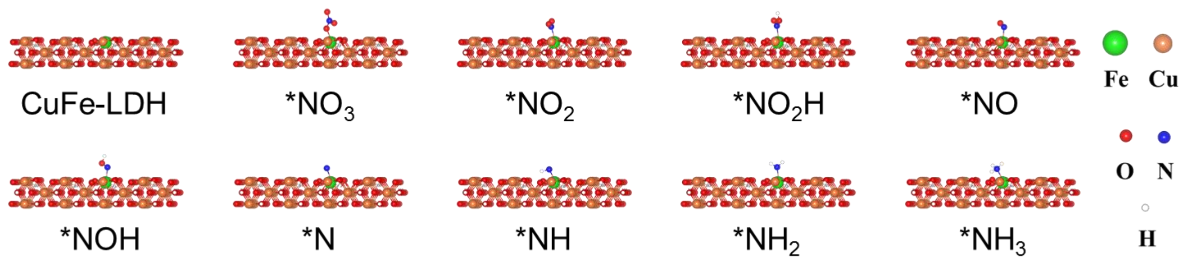

Figure S30. The adsorption model of each intermediate species on the CuFe-LDH surface at Cu active site.

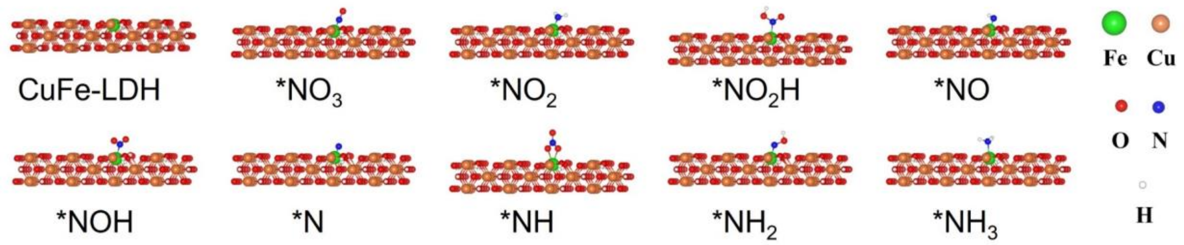

Figure S31. The adsorption model of each intermediate species on the CuFe-LDH surface at Fe active site.

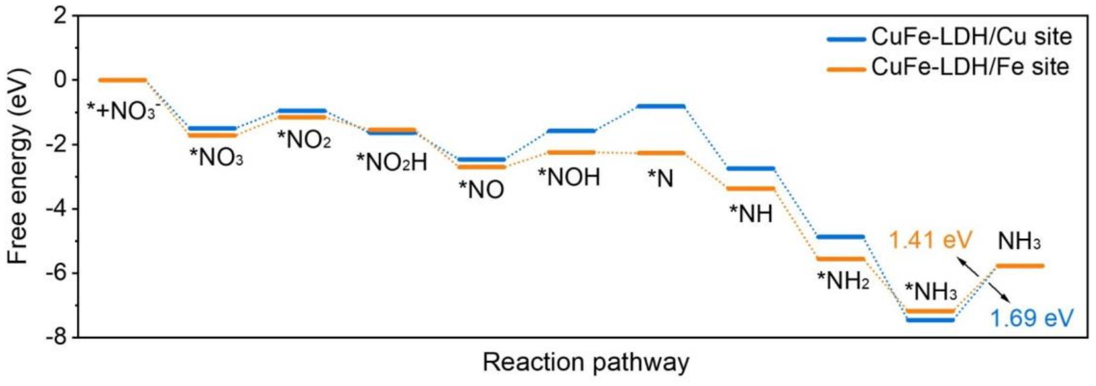

Figure S32. The free energy of CuFe-LDH at Fe active site and Cu active site, respectively.

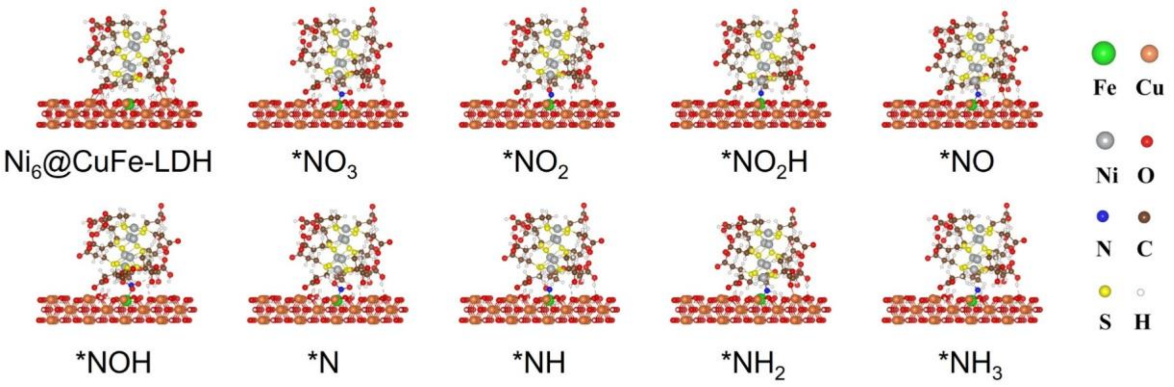

Figure S33. The adsorption model of each intermediate species on the Ni_6@CuFe-LDH surface at Fe active site.

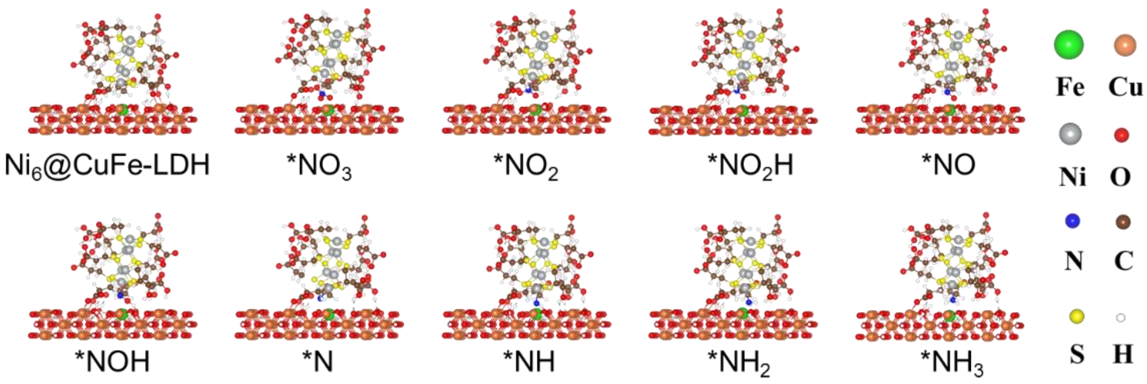

Figure S34. The adsorption model of each intermediate species on the Ni_6@CuFe-LDH surface at Ni active site.

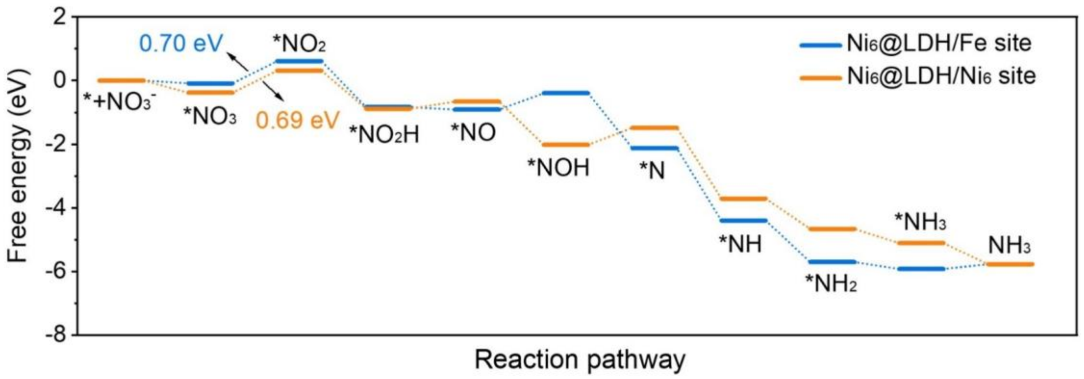

Figure S35 The free energy of Ni_6@CuFe-LDH at Fe active site and Ni cluster active site, respectively.

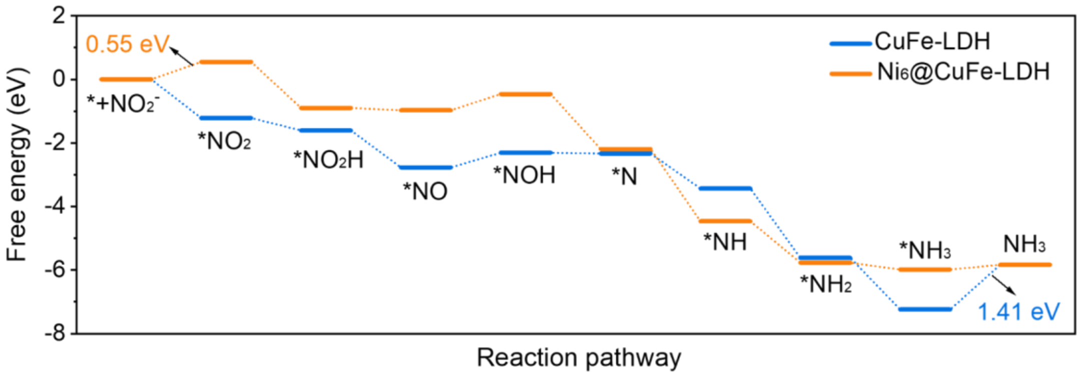

Figure S36 Free energy diagram for electrochemical NO_2^- reduction (NO_2RR) over CuFe-LDH and Ni_6@CuFe-LDH.

Note: For CuFe-LDH, NH_3 desorption remains the rate-determining step (RDS) with a high barrier of 1.41 eV (Fig S36), indicating overly strong binding between $*NH_3$ and the surface. In contrast, Ni_6@CuFe-LDH exhibits a significantly lower barrier for $*NH_3$ desorption, and the RDS shifts to the initial $* \rightarrow *NO_2$ step with a moderate barrier of 0.55 eV (Fig S36), reflecting improved intermediate desorption kinetics. This transition can be attributed to the Ni_6 cluster's ability to modulate the surface binding strength, effectively weakening $*NH_3$ adsorption and relieving product inhibition. As a result, the NO_2RR pathway becomes more favorable on Ni_6@CuFe-LDH, which explains the superior experimental NO_2RR performance shown in **Figure 3b-c**. Moreover, in the early stages of NO_2RR, the key intermediates such as $*NO_2$, $*NO_2H$, $*NO$, and $*NOH$ exhibit very strong adsorption on the CuFe-LDH surface. This strong binding may result in the accumulation of reactive species, particularly $*NO$, during the reduction of NO_2^-. As discussed in the main text, such intermediate accumulation can impede subsequent protonation steps and slow down the overall reaction kinetics, possibly leading to the formation of undesired byproducts. This not only validates the structural design of the Ni_6-modified system but also supports the conclusion that the enhanced NO_3RR activity originates from optimized reaction kinetics for the key NO_2 intermediate.

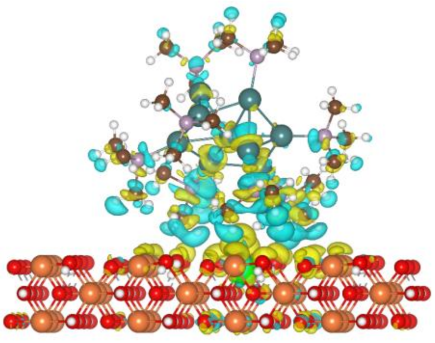

Figure S37. Charge density difference of Au_9@CuFe-LDH. (isosurface=0.001 electron/bohr^3)

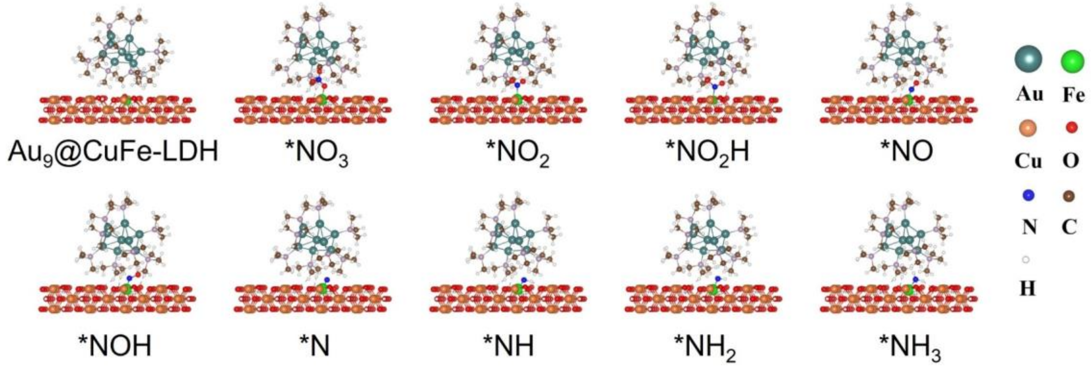

Figure S38. The optimal adsorption model of each intermediate species on the Au$_9$@CuFe-LDH surface.

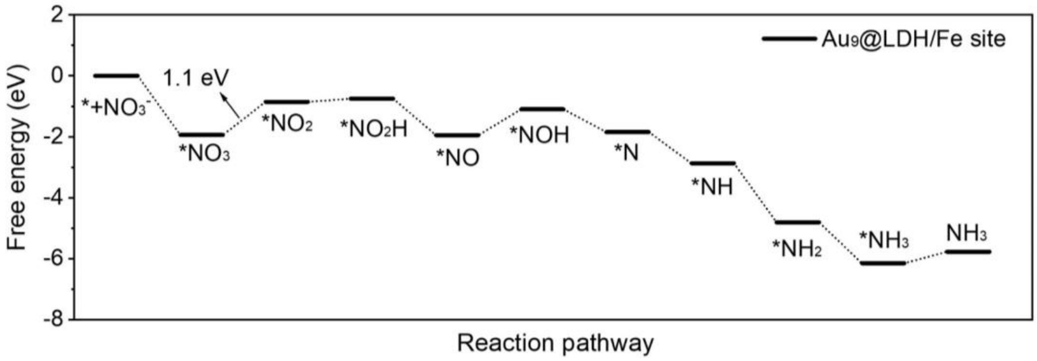

Figure S39. The free energy of Au$_9$@CuFe-LDH at Fe active site.

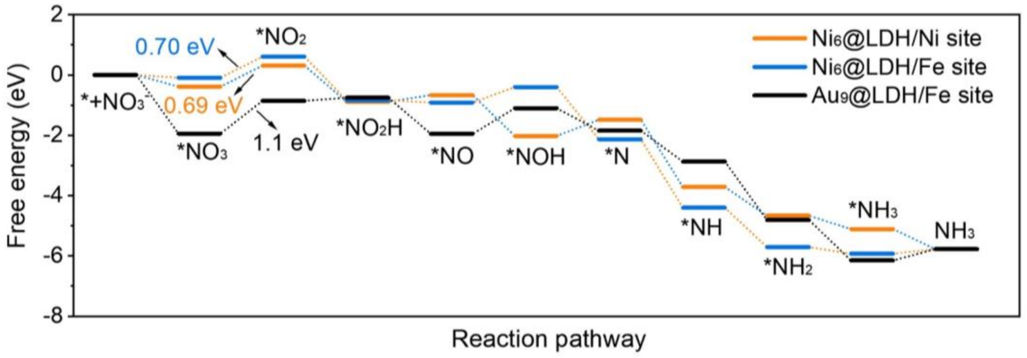

Figure S40. The free energy of Ni$_6$@CuFe-LDH at Ni active site and Fe active site and Au$_9$@CuFe-LDH at Fe site.

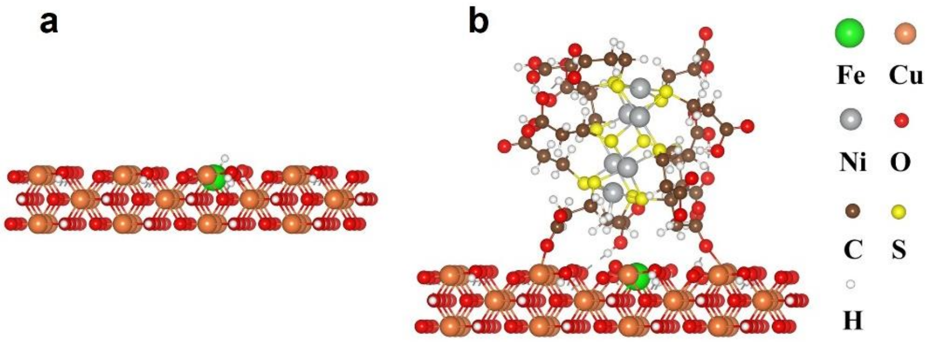

Figure S41. The optimal adsorption model of each intermediate species on CuFe-LDH and Ni$_6$@CuFe-LDH in HER.

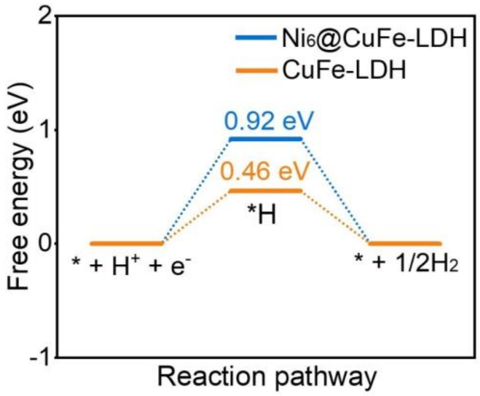

Figure S42. Free energy diagram for HER of CuFe-LDH and Ni_6@CuFe-LDH.

Table S1. XPS analysis of CuFe-LDH and Ni_6@CuFe-LDH.

	O_l%	O_v%	O_a%
CuFe-LDH	16.4	39.0	44.6
Ni_6@CuFe-LDH	36.2	48.1	15.7

Surface properties of CuFe-LDH and Ni_6@CuFe-LDH composite analyzed by XPS. Note: O_{total} is the sum of O_l, O_v, and O_a, and O_i% = O_i/O_{total}. Denotations: lattice oxygen (O_l), oxygen vacancy (O_v), and adsorbed oxygen (O_a).

Table S2. Fitting results of Ni K-edge EXAFS spectra for Ni_6@CuFe-LDH.

Samples	Path	*CNs*	*R* (Å)	σ^2 (Å^2)	$\triangle E_0$ (eV)	*R*-factor
	Ni-S	1.8	2.14	0.013		
Ni_6@CuFe-LDH	Ni-Ni	3.7	2.54	0.018	-1.82	0.018
	Ni-Cu/Fe	9.4	2.98	0.009		

Note: *CNs*, coordination number; *R*, distance between absorber and backscatter atoms; σ^2, Debye-Waller factors; $\triangle E_0$, inner potential correction that accounts for the difference in the inner potential between the sample and the references. *R*-factor: goodness of fit.

Table S3. Zeta potential of $Ni_6(MPA)_{12}$, CuFe-LDH and Ni_6@CuFe-LDH at pH=10.

	Zeta potential (mV)				
	1#	2#	3#	Mean	Standard Deviation
$Ni_6(MPA)_{12}$	-12.79	-11.46	-12.88	-12.38	0.80
CuFe-LDH	+30.54	+31.66	+31.55	+31.25	0.62
Ni_6@CuFe-LDH	+17.67	+17.70	+17.92	+17.76	0.14

Table S4. Compilation of recently reported NO_3RR activity over representative electrocatalysts.

Catalyst	Electrolyte	Formation Rate (NH_3)	FE (NH_3)	Potential (vs. RHE)	Ref.
**Ni_6@CuFe-LDH**	**1.0 M KOH + 0.1 M KNO_3**	**0.91 mmol mg^{-1} h^{-1} /0.55 mmol h^{-1} cm^{-2}**	**96.8%**	**-0.5 V**	**This work**
**Ni_6/CuFe-LDH**	**1.0 M KOH + 0.1 M KNO_3**	**0.76 mmol mg^{-1} h^{-1} /0.45 mmol h^{-1} cm^{-2}**	**97.3%**	**-0.5 V**	
**CuFe-LDH**	**1.0 M KOH + 0.1 M KNO_3**	**1.04 mmol mg^{-1} h^{-1} /0.62 mmol h^{-1} cm^{-2}**	**72.5%**	**-0.5 V**	
Cu_2O+Co_3O_4	0.1 M NaOH+0.1 M $NaNO_3$	0.75 mmol mg^{-1} h^{-1}	85.4%	–0.3 V	6
Cu (100)-rich rugged Cu-nanobelt (Cu-NBs-100)	1 M KOH+0.1 M KNO_3	0.65 mmol mg^{-1} h^{-1}	95.3%	−0.15 V	7
Fe-PPy SACs	0.1 M KOH + 0.1 M KNO_3	0.162 mmol h^{-1} cm^{-2}	98.4%	−0.7 V	8
Cu doped porous nitrogen-doped carbon single-atom catalyst (Cu-N-C SAC)	0.1 M KOH + 0.1 M KNO_3	4.5 mg h^{-1} cm^{-2}	84.7%	−1.0 V	9
Metastable phase Cu (MP-Cu)	1 M KOH + 0.05 M KNO_3^-	0.543 mmol h^{-1} cm^{-2}	99.8%	−0.3 V	10
Cu-doped Fe_3O_4	0.1 M KOH + 0.1 M KNO_3	7.18 mg h^{-1} cm^{-2}	100%	−0.6 V	11
Ni_3Fe–CO_3 LDH	1 M KOH + 5 mM KNO_3	1.261 mg h^{-1} cm^{-2}	96.8%	−0.2 V	12
Cu NPS@C	1 M KOH + 1 mM KNO_3	469.5 μg h^{-1} cm^{-2}	72%	−0.9 / −0.3 V	13
Ru_1Cu_{10}/rGO	1 M KOH + 100 mM KNO_3	0.38 mmol cm^{-2} h^{-1}	98%	−0.05 V	14
Cu/Cu_2O NWAs	0.5 M Na_2SO_4 + 200 ppm KNO_3	0.245 mmol cm^{-2} h^{-1}	95.8%	-0.85 V	15
Plasma-treated CuO (pCuO-5)	0.05 M H_2SO_4 + 0.05 M KNO_3	0.29 mmol cm^{-2} h^{-1}	80%	-0.6 V	16
Fe single-atom catalyst	0.10 M K_2SO_4 + 0.50 M KNO_3	0.46 mmol cm^{-2} h^{-1}	75%	−0.85 / −0.66 V	17
Atomic Ru sites loaded on nitrogenated carbon (Ru_{SA}-NC)	1 M KOH + 0.5 M KNO_3	0.15 mmol cm^{-2} h^{-1}	72.8%	-0.6 V	18
Hollow carbon-coated Cu NPs (HSCuAGB@C)	0.1 M KOH + 10 mM KNO_3	487.8 mmol g^{-1} h^{-1}	94.2%	-0.2 V	19
Cu clusters/TiO_{2-x}	0.5 M Na_2SO_4 + 500 ppm KNO_3	0.114 mmol h^{-1} mg^{-1}	81.34%	-0.75 V	20
Pd octohedra	0.1 M Na_2SO_4 + 0.1 M $NaNO_3$	0.549 mmol cm^{-2} h^{-1}	79.9%	-0.7 V	21
Ir nanotubes	0.1 M $HClO_4$ + 1 M $NaNO_3$	0.92 mmol mg^{-1} h^{-1}	84.7%	0.06 V	22

References

1. Grimme, S., Semiempirical GGA-type density functional constructed with a long-range dispersion correction. *J. Comput. Chem.* **2006,** *27*, 1787-1799.
2. Perdew, J. P.; Wang, Y., Pair-distribution function and its coupling-constant average for the spin-polarized electron gas. *Phys. Rev. B* **1992,** *46*, 12947-12954.
3. Mortensen, J. J.; Hansen, L. B.; Jacobsen, K. W., Real-space grid implementation of the projector augmented wave method. *Phys. Rev. B* **2005,** *71*, 035109.
4. Chadi, D. J., Special points for Brillouin-zone integrations. *Phys. Rev. B* **1977,** *16*, 1746-1747.
5. Grimme, S.; Antony, J.; Ehrlich, S.; Krieg, H., A consistent and accurate ab initio parametrization of density functional dispersion correction (DFT-D) for the 94 elements H-Pu. *J. Chem. Phys.* **2010,** *132*.
6. Zhang, J.; He, W.; Quast, T.; Junqueira, J. R. C.; Saddeler, S.; Schulz, S.; Schuhmann, W., Single-entity Electrochemistry Unveils Dynamic Transformation during Tandem Catalysis of Cu_2O and Co_3O_4 for Converting NO_3^- to NH_3. *Angew. Chem. Inter. Ed.* **2023,** *62*, e202214830.
7. Hu, Q.; Qin, Y.; Wang, X.; Wang, Z.; Huang, X.; Zheng, H.; Gao, K.; Yang, H.; Zhang, P.; Shao, M.; He, C., Reaction intermediate-mediated electrocatalyst synthesis favors specified facet and defect exposure for efficient nitrate–ammonia conversion. *Energy Environ. Sci.* **2021,** *14*, 4989.
8. Li, P.; Jin, Z.; Fang, Z.; Yu, G., A single-site iron catalyst with preoccupied active centers that achieves selective ammonia electrosynthesis from nitrate. *Energy Environ. Sci.* **2021,** *14*, 3522.
9. Yang, J.; Qi, H.; Li, A.; Liu, X.; Yang, X.; Zhang, S.; Zhao, Q.; Jiang, Q.; Su, Y.; Zhang, L.; Li, J.-F.; Tian, Z.-Q.; Liu, W.; Wang, A.; Zhang, T., Potential-Driven Restructuring of Cu Single Atoms to Nanoparticles for Boosting the Electrochemical Reduction of Nitrate to Ammonia. *J. Am. Chem. Soc.* **2022,** *144*, 12062.
10. Wen, W.; Yan, P.; Sun, W.; Zhou, Y.; Yu, X.-Y., Metastable Phase Cu with Optimized Local Electronic State for Efficient Electrocatalytic Production of Ammonia from Nitrate. *Adv. Funct. Mater.* **2023,** *33*, 2212236.
11. Wang, J.; Wang, Y.; Cai, C.; Liu, Y.; Wu, D.; Wang, M.; Li, M.; Wei, X.; Shao, M.; Gu, M., Cu-Doped Iron Oxide for the Efficient Electrocatalytic Nitrate Reduction Reaction. *Nano Lett.* **2023,** *23*, 1897.
12. Kim, K.-H.; Lee, H.; Huang, X.; Choi, J. H.; Chen, C.; Kang, J. K.; O'Hare, D., Energy-efficient electrochemical ammonia production from dilute nitrate solution. *Energy Environ. Sci.* **2023,** *16*, 663.
13. Song, Z.; Liu, Y.; Zhong, Y.; Guo, Q.; Zeng, J.; Geng, Z., Efficient Electroreduction of Nitrate into Ammonia at Ultralow Concentrations Via an Enrichment Effect. *Adv. Mater.* **2022,** *34*, 2204306.
14. Gao, W.; Xie, K.; Xie, J.; Wang, X.; Zhang, H.; Chen, S.; Wang, H.; Li, Z.; Li, C., Alloying of Cu with Ru Enabling the Relay Catalysis for Reduction of Nitrate to Ammonia. *Adv. Mater.* **2023,** *35*, 2202952.
15. Wang, Y.; Zhou, W.; Jia, R.; Yu, Y.; Zhang, B., Unveiling the Activity Origin of a Copper-based Electrocatalyst for Selective Nitrate Reduction to Ammonia. *Angew. Chem. Inter. Ed.* **2020,** *59*, 5350.
16. Daiyan, R.; Tran-Phu, T.; Kumar, P.; Iputera, K.; Tong, Z.; Leverett, J.; Khan, M. H. A.; Asghar Esmailpour, A.; Jalili, A.; Lim, M.; Tricoli, A.; Liu, R.-S.; Lu, X.; Lovell, E.; Amal, R., Nitrate reduction to ammonium: from CuO defect engineering to waste NO_x-to-NH_3 economic feasibility. *Energy Environ. Sci.* **2021,** *14*, 3588.
17. Wu, Z.-Y.; Karamad, M.; Yong, X.; Huang, Q.; Cullen, D. A.; Zhu, P.; Xia, C.; Xiao, Q.; Shakouri, M.; Chen, F.-Y.; Kim, J. Y.; Xia, Y.; Heck, K.; Hu, Y.; Wong, M. S.; Li, Q.; Gates, I.; Siahrostami, S.; Wang, H., Electrochemical ammonia synthesis via nitrate reduction on Fe single atom catalyst. *Nat. Commun.* **2021,** *12*, 2870.
18. Ke, Z.; He, D.; Yan, X.; Hu, W.; Williams, N.; Kang, H.; Pan, X.; Huang, J.; Gu, J.; Xiao, X., Selective NO_x^- Electroreduction to Ammonia on Isolated Ru Sites. *ACS Nano* **2023,** *17*, 3483.
19. Hu, Q.; Qin, Y.; Wang, X.; Zheng, H.; Gao, K.; Yang, H.; Zhang, P.; Shao, M.; He, C., Grain Boundaries Engineering of Hollow Copper Nanoparticles Enables Highly Efficient Ammonia Electrosynthesis from Nitrate. *CCS Chem.* **2022,** *4*, 2053.
20. Zhang, X.; Wang, C.; Guo, Y.; Zhang, B.; Wang, Y.; Yu, Y., Cu clusters/TiO_{2-x} with abundant oxygen vacancies for enhanced electrocatalytic nitrate reduction to ammonia. *J. Mater. Chem. A* **2022,** *10*, 6448.

21. Han, Y.; Zhang, X.; Cai, W.; Zhao, H.; Zhang, Y.; Sun, Y.; Hu, Z.; Li, S.; Lai, J.; Wang, L., Facet-controlled palladium nanocrystalline for enhanced nitrate reduction towards ammonia. *J. Colloid Interface Sci.* **2021,** *600*, 620.
22. Zhu, J.-Y.; Xue, Q.; Xue, Y.-Y.; Ding, Y.; Li, F.-M.; Jin, P.; Chen, P.; Chen, Y., Iridium Nanotubes as Bifunctional Electrocatalysts for Oxygen Evolution and Nitrate Reduction Reactions. *ACS Appl. Mater. Interfaces* **2020,** *12*, 14064.
